# Neural ensembles that encode nocifensive mechanical and heat pain in mouse spinal cord

**DOI:** 10.1038/s41593-025-01921-6

**Published:** 2025-03-24

**Authors:** Ming-Dong Zhang, Jussi Kupari, Jie Su, Kajsa A. Magnusson, Yizhou Hu, Laura Calvo-Enrique, Dmitry Usoskin, Gioele W. Albisetti, Mikaela M. Ceder, Katharina Henriksson, Andrew D. Leavitt, Hanns Ulrich Zeilhofer, Tomas Hökfelt, Malin C. Lagerström, Patrik Ernfors

**Affiliations:** 1 Department of Medical Biochemistry and Biophysics, Division of Molecular Neurobiology, Karolinska Institutet, Stockholm, Sweden; 2 Department of Immunology, Genetics and Pathology, Uppsala University, Uppsala, Sweden; 3 Institute of Pharmacology and Toxicology, University of Zurich, Zurich, Switzerland; 4 Institute of Pharmaceutical Sciences, Swiss Federal Institute of Technology (ETH) Zurich, Zurich, Switzerland; 5 Department of Medicine, University of California, San Francisco, CA USA; 6 Department of Laboratory Medicine, University of California, San Francisco, CA USA; 7 Department of Neuroscience, Karolinska Institutet, Stockholm, Sweden; 8 Present Address: Department of Cell Biology and Pathology, Instituto de Neurociencias de Castilla y León (INCyL), Universidad de Salamanca, Salamanca, Spain

**Keywords:** Chronic pain, Neural circuits

## Abstract

Acute pain is an unpleasant experience caused by noxious stimuli. How the spinal neural circuits attribute differences in quality of noxious information remains unknown. By means of genetic capturing, activity manipulation and single-cell RNA sequencing, we identified distinct neural ensembles in the adult mouse spinal cord encoding mechanical and heat pain. Reactivation or silencing of these ensembles potentiated or stopped, respectively, paw shaking, lifting and licking within but not across the stimuli modalities. Within ensembles, polymodal *Gal*^+^ inhibitory neurons with monosynaptic contacts to A-fiber sensory neurons gated pain transmission independent of modality. Peripheral nerve injury led to inferred microglia-driven inflammation and an ensemble transition with decreased recruitment of *Gal*^+^ inhibitory neurons and increased excitatory drive. Forced activation of *Gal*^+^ neurons reversed hypersensitivity associated with neuropathy. Our results reveal the existence of a spinal representation that forms the neural basis of the discriminative and defensive qualities of acute pain, and these neurons are under the control of a shared feed-forward inhibition.

## Abstract (web summary)

Zhang et al. identify unimodal neural representations in the spinal cord of cutaneous mechanical and heat stimuli gated by a shared feed-forward local inhibitory neuron type and a neural transition during neuropathy, with increased excitatory drive.

## Main

Detection and response to intense mechanical force and heat enables protection from what can cause injury. Withdrawal reflexes and the unpleasant experience of pain are initiated by the activation of specialized primary afferent sensory neurons with peripheral termini in the skin and central termini in the spinal cord. Within the spinal cord, the incoming information is processed before it is relayed to higher brain regions. A local neuronal network (that is, ensemble) contacted by nociceptor terminals consists of excitatory and inhibitory interneurons as well as projection neurons. How spinal cord neurons transform nociceptive information into a neural computation representing the discriminative and defensive qualities of pain remains unclear^1^. Several models have been proposed for encoding nociceptive information carried by primary afferent sensory neurons in the spinal cord, including dedicated molecular neuron types, necessity of temporal summation or patterns of firing and various combinations of these ideas^1–3^. Although in activity recordings, most spinal projection neurons display a polymodal response, some neurons respond only to noxious mechanical stimuli or heat^4–7^, illustrating the existence of a neural representation of mechanical and heat sensations. However, functional manipulation of populations of excitatory spinal neurons defined by neurochemical markers revealed these as polymodal, affecting both mechanical and thermal nociception or with marginal modality-selective preference^1,8–11^.

Embedded within these circuits, inhibitory neurons play a critical role^12^, as was originally proposed in the gate control theory of pain^13^, and reduced inhibitory activity induces allodynia and hyperalgesia across all sensory modalities and pain states similar to those associated with chronic pain^14^. Such disinhibition increases excitability of *Tacr1*-expressing lamina I projection neurons known to transmit noxious information from the spinal cord to the brain^15–17^. Thus, spinal inhibitory neurons maintain appropriate activity levels in the neuronal circuits by regulating afferent sensory information on the way to output projection neurons. Recently, assessing function of neurons that express *Pdyn* and *Pvalb* marker genes shows that these neurons prevent touch inputs from activating pain circuits. Consistently, disinhibition leads to a marked reduction in the threshold for the mechanically evoked withdrawal reflex^1,8,18^. Thus, little is known beyond neural mechanisms for mechanical threshold detection initiating a withdrawal reflex.

Activity-dependent expression of the *Fos* gene in the spinal cord marks neural activity caused by peripheral noxious stimuli^19^. Although efforts have been made to decode these activated neurons in the spinal cord using different genetic strategies, there has been limited progress^9,20,21^. Single-cell RNA sequencing (scRNA-seq) has revealed a marked transcriptional heterogeneity, including up to 30 excitatory and inhibitory neuron types in the dorsal horn of the spinal cord^22–24^. A molecular atlas of spinal neurons and genetic strategies that captures active neurons with high temporal resolution opens the door for functional and molecular studies that can provide insights into the neural substrates that represent noxious information in the spinal cord. Here, we identify the neural ensembles in the spinal cord that encode the defensive nocifensive dimension of noxious mechanical and heat stimuli.

## Results

### Parallel spinal ensembles encoding mechanical and heat stimuli

**Fig. 1:**
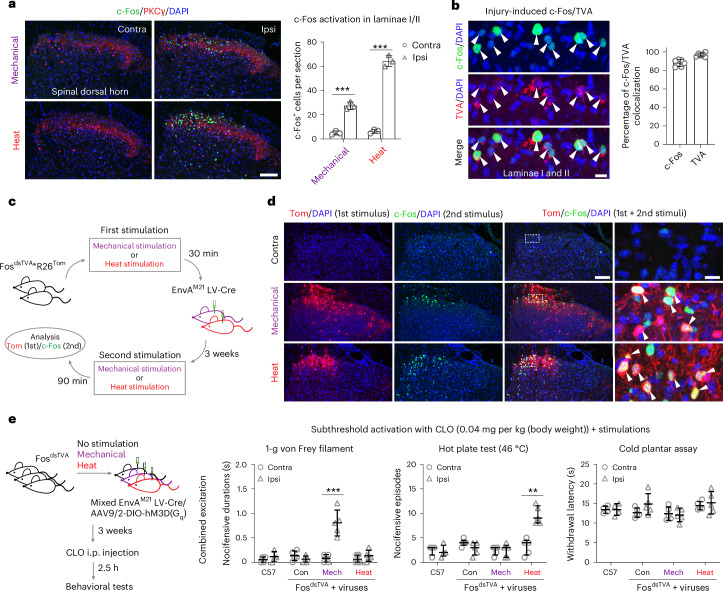
Capturing ensembles encoding mechanical and heat pain in the spinal dorsal horn. **a**, Left: representative images of c-Fos and PKCγ immunohistochemistry in the medial spinal dorsal horn after noxious stimulations. Right: auantification of c-Fos expression is shown (*n* = 6 mice). **b**, Left: colocalization of destabilized TVA and c-Fos in the spinal cord of Fos^dsTVA^*R26^Tom^ mice induced by skin incision and/or nerve injury. Right: colocalization between c-Fos and TVA neurons was quantified (*n* = 6 mice). Arrowheads indicate colocalization. **c**, Schematic of the validation experiment for the same type of stimulus; LV-Cre, lenti-Cre virus. **d**, Representative immunohistochemical images show ensembles activated and labeled with Tomato (Tom) and c-Fos in the spinal dorsal horn as illustrated in **c** (*n* = 3 mice per group). Right: high-magnification images from the dashed white boxes show the colocalization indicated by the arrowheads. **e**, Left: schematic of the gain-of-function study of ensembles infected with AAV9/2-DIO-hM3D(G_q_) through treatment with subthreshold clozapine (CLO) combined with application of a peripheral stimulus; i.p., intraperitoneal; Mech, mechanical. Right, nocifensive durations/episodes for the 1-g von Frey filament test and hot plate test (46 °C) and withdrawal latency for the cold plantar assay under subthreshold activation of mechanical or heat ensembles (*n* = 5 for mechanical and *n* = 5 for heat); Con, Fos^dsTVA^ mice (*n* = 5) injected with virus without stimulation; C57, wild-type mice (*n* = 4); Contra, contralateral; Ipsi, ipsilateral; scale bars, 100 μm in **a** and **d** and 25 μm in **b** and magnified image in **d**. Data are expressed as mean ± s.d. Differences between contralateral and ipsilateral sides from the different groups were analyzed by ordinary one-way analysis of variance (ANOVA), followed by a Bonferroni multiple comparisons test (adjusted *P* < 0.0001 for both mechanical and heat groups in **a**; adjusted *P* values from left: *P* > 0.9999, *P* > 0.9999, *P* < 0.0001 and *P* > 0.9999 for the 1-g von Frey filament test and *P* > 0.9999, *P* *=* 0.3161, *P* > 0.9999 and *P* > 0.9999 for the cold plantar test in **e**). The nocifensive episodes for the hot plate test in **e** are presented as median with interquartile range, and differences between contralateral and ipsilateral sides from each group were analyzed by Mann–Whitney test (two-tailed *P* values from left: *P* = 0.5714, *P* *=* 0.2063, *P* > 0.9999 and *P* *=* 0.0079); ***P < 0.001 and **P < 0.01. Source data

**Extended Data Fig. 1:**
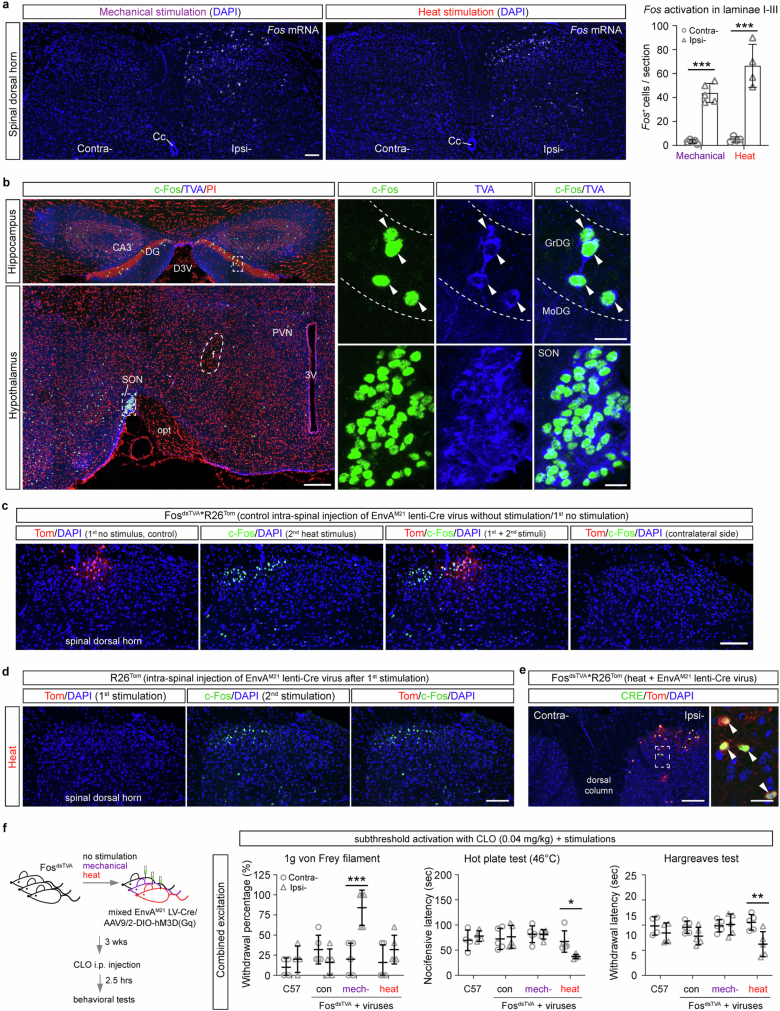
*Fos* activation, specificity of Fos^dsTVA^ mice and modulation of active neurons labelled by *Fos* expression. **a**, Representative images of *Fos* mRNA in spinal dorsal horn induced by noxious stimuli and quantification for *Fos*^+^ neurons (n = 5 for mechanical and 4 for heat). Cc, central canal. **b**, Representative images of co-expression between c-Fos and TVA in hippocampus and hypothalamus from Fos^dsTVA^ mice (n = 2) counterstained with propidium iodide (PI). High magnification images show dentate gyrus (DG) and supraoptic nucleus (SON) from the insets. GrDG, granule cell layer of DG; MoDG, molecular DG; D3V, dorsal 3rd ventricle; opt, optic tract; PVN, paraventricular nucleus of hypothalamus. Arrowheads indicate co-localization. **c**, Representative images show a limited off-target labeling (Tom) of EnvA^M21^ lenti-Cre virus along injection tract induced by surgery (n = 2). **d**, In control R26^Tom^ mice pairing Tomato (1^st^ stimulus) and c-Fos (2^nd^ stimulus) revealed no Tom^+^ signal (n = 2). **e**, Representative images examining in Fos^dsTVA^*R26^Tom^ mice Cre expression in Tom^+^ cells following heat stimulus (n = 4). **f**, Left, schematic diagram for the functional study of gain of function for ensembles infected by AAV9/2-DIO-hM3D(Gq) virus. Right, 1g von Frey filament test, hot plate test (46°C) and cold plantar assay under subthreshold activation of mechanical or heat ensembles (0.04 mg/kg clozapine, n = 19). Con: Fos^dsTVA^ mice injected virus without stimulation; C57: wild type mice; Contra-: contralateral; Ipsi-: ipsilateral. DAPI was used as a counterstaining. Scale bars = 100 μm in (**a**, **c** and **d**), 200 μm for the overview and 20 μm for high magnification in (**b**), 100 μm the overview and 20 μm for the inset in (**e**). Data are expressed as mean ± SD. Differences between contra- and ipsi- from different groups were analyzed by ordinary one-way ANOVA followed by Bonferroni’s multiple comparisons test (**a**, **f**). Adjusted *P* < 0.0001 for both stimulations in (**a**), values from left > 0.9999, 0.7309, < 0.0001 and 0.7309 for 1g von Frey filament, > 0.9999, > 0.9999, > 0.9999 and 0.0397 for hot plate test, > 0.9999, 0.5146, > 0.9999 and 0.0026 for Hargreaves test in (**f**). *** indicates *P* < 0.001, ** indicates *P* < 0.01 and * indicates *P* < 0.05. Source data

Robust and reproducible stimulation paradigms for activation of spinal neurons by peripheral mechanical or heat stimuli were established. Medial superficial layers of the corresponding lumbar spinal dorsal horn that receive inputs from the hind paw contained *Fos*^+^ (mRNA) and c-Fos^+^ (protein) neurons (Extended Data Fig. 1a and Fig. 1a). To identify and access these mechanically or heat-active neurons for manipulation, we used the stimulus-coupled transcription technology capturing activated neuronal ensembles (CANE), which affords very high temporal resolution^25^. This technique is based on rapid expression and degradation of avian tumor virus A (TVA) from the *Fos* locus in excited neurons. TVA is the receptor for the avian sarcoma and leukosis virus EnvA glycoprotein. Lentivirus and rabies virus can be EnvA pseudotyped and thus made to infect only cells that express TVA. For the CANE technique, a mouse line (Fos^dsTVA^) in which *Fos* drives the expression of destabilized TVA was generated. Therefore, if introduction of an EnvA pseudotyped lentivirus driving Cre recombinase expression in the spinal cord in Fos^dsTVA^ mice parallels the expression of destabilized TVA, only these very recently excited neurons get infected and express Cre. Specificity of the Fos^dsTVA^ mouse strain was validated by colocalization of injury-induced c-Fos and TVA expression in superficial layers of the spinal cord and brain (Fig. 1b and Extended Data Fig. 1b). Fos^dsTVA^ mice crossed with Rosa26^Tomato^ reporter mice (Fos^dsTVA^*R26^Tom^) were used to ascertain the accuracy of captured active neurons in the spinal cord. Fos^dsTVA^*R26^Tom^ mice were subjected to a mechanical or heat stimulus and intraspinally injected with Cre-expressing EnvA pseudotyped lentivirus (EnvA^M21^ lenti-Cre virus). Three weeks later, Tomato reporter expression initiated at the first stimulation (‘captured’ neurons) was compared to c-Fos expression induced by a second stimulation (Fig. 1c,d). The overlaps were 51.78 ± 4.69% and 68.59 ± 4.54% for mechanical and heat stimuli, respectively (Fig. 1d), whereas control EnvA^M21^ lenti-Cre virus injection with no stimulus led to limited capture and only along the injection tract (12.0% overlap along injection tracts; Extended Data Fig. 1c). Tomato expression was absent in control R26^Tom^ mice that received EnvA^M21^ lenti-Cre virus, and, furthermore, Tomato expression was only observed in neurons infected by the EnvA^M21^ lenti-Cre virus through TVA (Tomato and Cre double-expressing neurons, 96.10 ± 3.15%; Extended Data Fig. 1d,e).

We next examined the importance of the mechanical and heat ensembles for behaviors evoked by noxious stimuli and whether subthreshold activation resulted in cross-modality effects using Fos^dsTVA^ mice intraspinally injected with a mixture of EnvA^M21^ lenti-Cre and AAV9/2-hEF1a-DIO-hM3D(G_q_)-mCherry. Nocifensive behaviors were defined as paw shaking, lifting/guarding or licking, whereas flexor withdrawal was analyzed separately. Although nocifensive behavior is defensive and perhaps soothes suffering, reflexive withdrawal behavior measured by mechanical withdrawal threshold and withdrawal latency to heat/cold serves to protect or limit injury^10^. Intraspinal virus administration coincident with a mechanical or heat stimulus led to activity-dependent, temporally and spatially controlled DNA recombination and hM3D(G_q_) DREADD expression. Because mice were anesthetized, hM3D(G_q_) expression does not reflect pain behavior-activated neurons. A subthreshold dose of clozapine, which itself resulted in no/rare nocifensive behaviors in wild-type control (C57BL/6), control Fos^dsTVA^ (no stimulation but with virus injected) or Fos^dsTVA^ mice (stimulation and virus injected), was titrated (0.04 mg per kg (body weight), 2.5–4.0 h after injection). When subthreshold activation was combined with weak natural stimuli normally without behavioral response by itself (1-g von Frey and 46 °C), a robust nocifensive behavior was observed within modality and without any effects across modalities, indicating that these ensembles encode the discriminative quality of acute pain and intensity (Fig. 1e). Subthreshold activation also resulted in moderate effects on withdrawal reflex behavior (Extended Data Fig. 1f), suggesting that forced activation can access withdrawal reflex circuits.

**Fig. 2:**
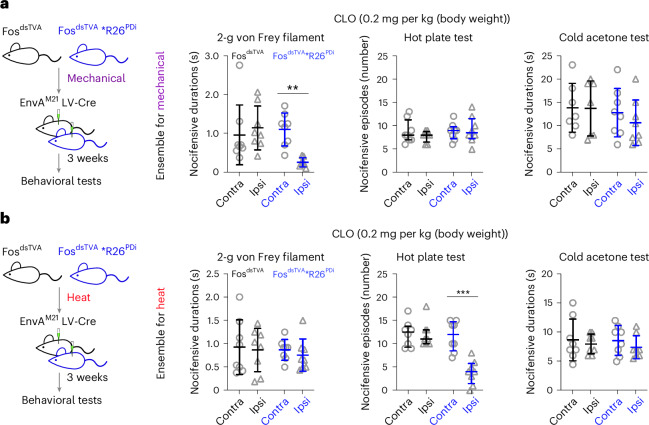
Chemogenetic inhibition of ensembles encoding mechanical and heat pain in the spinal cord. **a**,**b**, Experimental paradigm for chemogenetic inhibition of ensembles that encode mechanical pain (**a**) and heat pain (**b**) in the spinal dorsal horn with intraspinal-injected pseudotyped EnvA^M21^ lenti-Cre virus in Fos^dsTVA^*R26^PDi^ and Fos^dsTVA^ (control) mice (*n* = 32 mice, 8 mice per group). Nocifensive behavior (durations or episodes) was assessed with a 2-g von Frey filament test (mechanical), hot plate test (heat; 50 °C) and cold acetone test (cold) 60 min after intraperitoneal administration of clozapine (0.2 mg per kg (body weight)); Contra, contralateral; Ipsi, ipsilateral. Data are expressed as mean ± s.d. for nocifensive durations and median with interquartile range for nocifensive episodes. Nocifensive duration differences between contralateral and ipsilateral sides from different groups were analyzed by ordinary one-way ANOVA followed by a Bonferroni multiple comparisons test (adjusted *P* values from left in **a**: *P* = 0.9971 and *P* *=* 0.0067 for the 2-g von Frey filament test and *P* > 0.9999 and *P* > 0.9999 for the cold acetone test; adjusted *P* values from left in **b**: *P* > 0.9999 and *P* > 0.9999 for the 2-g von Frey filament test and *P* > 0.9999 and *P* > 0.9999 for the cold acetone test). The nocifensive episode differences between contralateral and ipsilateral sides from each group were analyzed by Mann–Whitney test (two-tailed; *P* = 0.6908 for Fos^dsTVA^ and *P* = 0.938 for Fos^dsTVA^*R26^PDi^ in **a** and *P* = 0.9764 for Fos^dsTVA^ and *P* = 0.0005 for Fos^dsTVA^*R26^PDi^ in **b**); ***P < 0.001 and **P < 0.01. Source data

**Extended Data Fig. 2:**
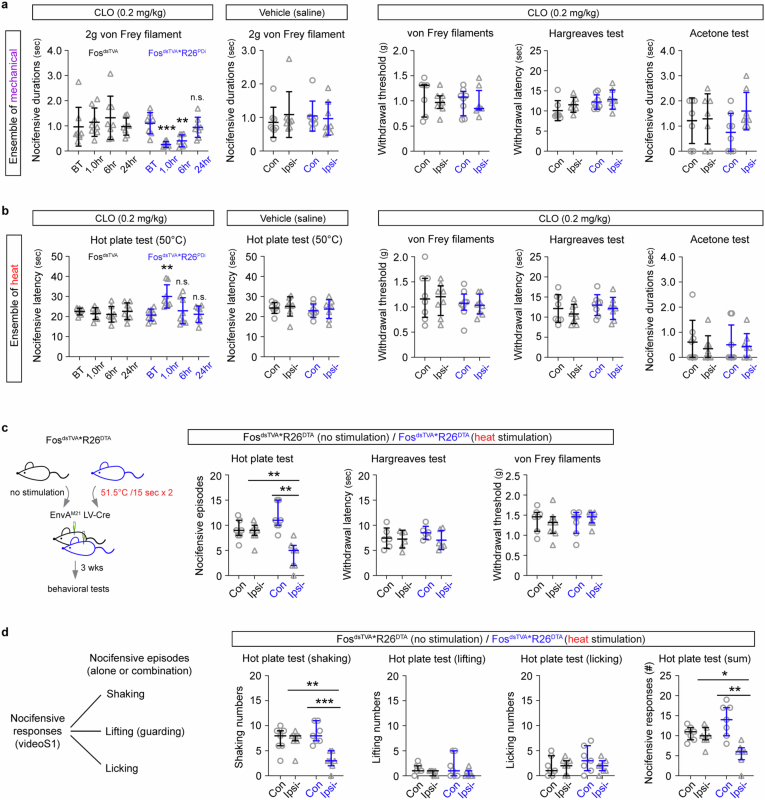
Inhibition of ensembles coding for mechanical or heat pain in spinal cord with chemogenetics or ablation of ensembles for heat with R26^DTA^ mice. Spinal ensembles were captured with EnvA^M21^ lenti-Cre virus in Fos^dsTVA^*R26^PDi^ mice (**a** and **b**). **a**, 2g von Frey filament test at different time points after application of clozapine (i.p.). Saline was used as the vehicle after 1.0 hr injection. Von Frey filaments test (non-noxious mechanical), Hargreaves test (thermo test) and acetone test (non-noxious cold/cool) were assessed after 60 min of application of clozapine (n = 16 mice, 8 mice/group, Fos^dsTVA^ mice as controls). Some tests only included 7 control mice. BT, basal threshold. **b**, Heat stimulation was assessed with hot plate test at different time points after application of clozapine (saline as vehicle, n = 16 mice, 8 mice/group). Von Frey filaments test, Hargreaves test and acetone test were assessed as in (**a**). **c**, Left: schematic diagram for ablation of spinal heat ensemble. Right: hot plate test (50°C), Hargreaves test and von Frey filaments test were assessed (n = 14 mice, 7 mice/group). Hargreaves test only used 6 mice from each group. **d**, Left: illustration of nocifensive responses and nocifensive episodes. Right: quantification of individual nocifensive response component and sum of the responses for hot plate test in (**c**). Data for durations and latency are expressed as mean ± SD and the rest are expressed as median with interquartile range. Differences of parametric data for different time points from ipsilateral and differences between contra- and ipsi- within the group were analyzed by ordinary one-way ANOVA followed by Bonferroni’s multiple comparisons test. The differences for nonparametric data between contra- and ipsi- from each group or ipsi- sides from different groups were analyzed by Mann-Whitney test (two-tailed). Adjusted *P* values from left 0.9321, 0.6483, > 0.9999 for Fos^dsTVA^ mice and 0.0004, 0.0052, > 0.9999 for Fos^dsTVA^*R26^PDi^ mice (time points, **a**), 0.7734 and > 0.9999 for vehicle (**a**), *P* = 0.4779 and 0.6623 for von Frey filaments, adjusted *P* values 0.4333 and > 0.9999 for Hargreaves, > 0.9999 and 0.1148 for acetone test (**a**). Adjusted *P* > 0.9999 from different time points for Fos^dsTVA^ mice and 0.003, > 0.9999, > 0.9999 for Fos^dsTVA^*R26^PDi^ mice (**b**), > 0.9999 and > 0.9999 for vehicle (**b**), *P* = 0.8591 and 0.6291 for von Frey filaments, adjusted *P* values 0.7114 and > 0.9999 for Hargreaves, 0.8920 and > 0.9999 for acetone test (**b**). *P* = 0.8328 for control, 0.0012 for mice with stimulation and 0.0076 between ipsilateral sides for hot plate test (**c**), adjusted *P* values from left > 0.9999, 0.3321, 0.3287 and 0.6358 for Hargreaves and von Frey filaments (**c**). *P* = 0.7855 for control mice, 0.0006 for mice with stimulation and 0.0047 between ipsilateral sides for shaking numbers (**d**), *P* = 0.204, 0.4167 and 0.5921 for lifting numbers, *P* = 0.8217, 0.3473 and 0.8013 for licking numbers, *P* = 0.5798, 0.0012 and 0.0105 for summed numbers (**d**). *** indicates *P* < 0.001, ** indicates *P* < 0.01, * indicates *P* < 0.05 and n.s. means no significance. Source data

To establish the causal role, we used the inhibitory hM4Di-DREADD, the expression of which is Cre dependent in the Fos^dsTVA^*R26^PDi^ mouse strain. We first captured and expressed hM4Di-DREADD in the spinal ensemble activated by noxious mechanical stimuli. Silencing the ensemble 3 weeks later led to a pronounced loss of nocifensive behavior to pricking pain (2-g von Frey filament) with a temporal effect corresponding to peak plasma concentration and clearance of clozapine. By contrast, nocifensive behavior to noxious heat (50 °C hot plate) or cold (prechilled acetone) was unchanged (Fig. 2a and Extended Data Fig. 2a). Furthermore, there was no effect on withdrawal latency to heat or cold or thresholds to punctate mechanical force evoked by von Frey filaments (Extended Data Fig. 2a). Thus, this mechanical ensemble of spinal neurons transforms afferent nociception information into a signal necessary for nocifensive behavior to noxious mechanical stimuli.

We next captured and expressed hM4Di in spinal neurons activated by noxious heat. Silencing the heat ensemble did not lead to any changes in nocifensive behavior in response to noxious pricking or cold; however, a near complete absence of noxious heat nocifensive behavior was observed, with an effect that corresponded to peak plasma concentration and clearance of clozapine (Fig. 2b and Extended Data Fig. 2b)^26^. There was no effect on withdrawal latency to heat or cold or withdrawal thresholds to punctate mechanical force evoked by von Frey filaments (Fig. 2b and Extended Data Fig. 2b). Intraspinal injection of EnvA^M21^ lenti-Cre virus did not affect behavior in control Fos^dsTVA^ mice, and clozapine alone had no effect on contralateral limb behavior in any of the experiments (Fig. 2a,b). Similar results were obtained for mechanical and heat pain when ablating instead of silencing the heat ensemble in Fos^dsTVA^*R26^DTA^ mice (Extended Data Fig. 2c). Shaking numbers contributed the most to the nocifensive response count that included shaking, lifting and licking behaviors in the hot plate test (Extended Data Fig. 2d). Collectively, these results show the existence of parallel ensembles in the spinal cord that are required for nocifensive mechanical and heat behavior within, but not across, noxious stimulus modalities.

### Molecularly defined ensembles

**Fig. 3:**
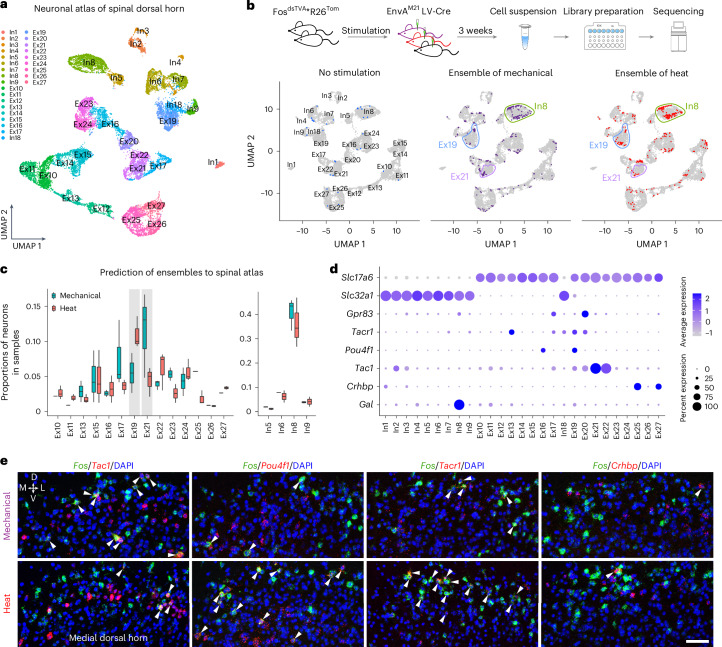
Decoding ensembles encoding mechanical and heat pain with scRNA-seq. **a**, Uniform manifold approximation and projection (UMAP) composed of the scRNA-seq data from 18,590 spinal dorsal horn neurons from adult mice (BAF53b-Cre*R26^Tom^, 12–20 weeks old, *n* = 8 mice) representing 27 clusters with 10 inhibitory (In1–In9 and In18) and 17 excitatory (Ex10–Ex17 and Ex19–Ex27) cell types in the adult spinal neuronal reference atlas. **b**, Top: experimental paradigm for scRNA-seq of ensembles encoding mechanical and heat pain. Bottom: sequenced neurons from control, mechanical and heat ensembles were plotted on the reference atlas by label transfer (*n* = 32 mice, including 8 mice for control ensembles, 12 mice for mechanical ensembles and 12 mice for heat ensembles). Modality-specific or shared cell types are highlighted with circles. **c**, Percentages of cell types from mechanical and heat ensembles derived from **b** were plotted for comparison (*n* = 4 mice per group, three groups per condition). Shades of gray highlight modality-specific cell types (scCODA, false discovery rate < 0.2). For the box plots, the center line represents the median, the box limits represent the top and bottom quartiles, and the whiskers represent the minimum and maximum. **d**, Dot plot of the expression of marker genes for excitatory neurons (*Slc17a6* (vGLUT2)), inhibitory neurons (*Slc32a1* (VGAT)), projection neurons (*Gpr83* and *Tacr1*) and specific cell types (*Pou4f1* (Ex19/Ex16), *Tac1* (Ex21/Ex22), *Crhbp* (Ex25/Ex27) and *Gal* (In8)). **e**, Representative images showing mRNA coexpression of *Tac1*, *Pou4f1*, *Tacr1* or *Crhbp* with *Fos* in the medial spinal dorsal horn from mice subjected to mechanical or heat stimulation (*n* = 4 or 5 mice for mechanical stimulation and 4 mice for heat stimulation). DAPI was used as a nuclear counterstain. Arrowheads indicate colocalization; scale bar, 50 μm; D, dorsal; M, medial; L, lateral; V, ventral. Source data

**Extended Data Fig. 3:**
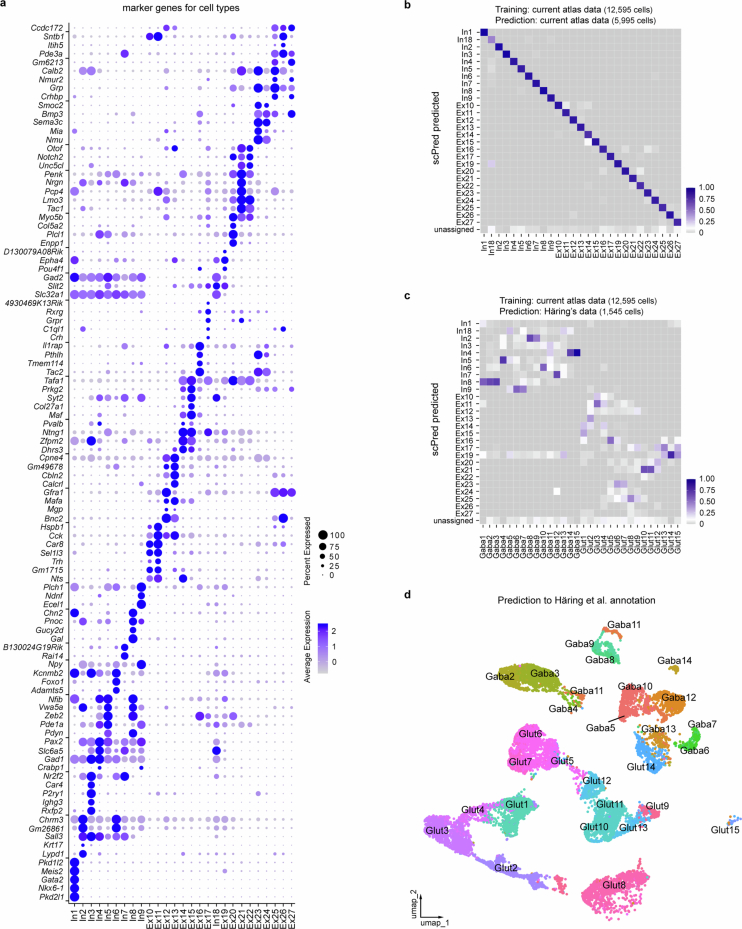
Expression of cluster marker genes of spinal dorsal horn neuronal atlas and comparison with Häring’s annotation. **a**, Dot Plot of top 5 genes for each cluster of the atlas. **b**, Heatmap of prediction of neurons from current atlas dataset with scPred to their known identity in the atlas. **c**, Heatmap of prediction of Häring’s dataset to current atlas annotation with scPred. **d**, UMAP of neurons from current atlas with predicted Häring’s annotation by Seurat label transfer.

To determine the neural basis for encoding mechanical and heat pain signals in the spinal cord, we used scRNA-seq to identify noxious mechanical and heat ensembles. Prior single-cell transcriptomic studies of the mouse spinal dorsal horn were hampered by relatively few whole sequenced cells from young animals, reduced quality due to nuclei sequencing or loss of variability due to integration of whole-cell and single-nucleus datasets across different RNA-seq platforms^22–24^. Identification of mechanical and heat ensembles depends on the quality of the spinal cord atlas and the age of the animals. We performed whole-cell sequencing of lumbar spinal dorsal horn neurons from adult BAF53b-Cre*R26^Tom^ mice, where all neurons were labeled with Tomato (Supplementary Fig. 1a–c). The neuronal atlas contained 18,590 neurons (4,926 genes detected/median), which clustered into 10 inhibitory (In1–In9 and In18) and 17 excitatory (Ex10–Ex17 and Ex19–Ex27) neuron types (Fig. 3a and Supplementary Fig. 2a–e). This atlas provided a more powered dataset to explore gene expression than prior annotations^22,24^, with a number of unique markers defining each neuron type (Extended Data Fig. 3a), and also included small cell clusters, for example, *Pkd2l1*^+^ In1 neurons (*Slc32a1*^+^*Scl6a5*^−^, cerebrospinal fluid-contacting neurons; Extended Data Fig. 3a) in lamina X^27^. A machine learning classifier (scPred)^28^ trained with about two-thirds of the neurons was tested on the remaining neurons with a prediction accuracy of 91.94% (Extended Data Fig. 3b). The classifier showed a high prediction accuracy to Häring’s^22^ atlases, with most cells identified in both (Extended Data Fig. 3c,d). The increased sequencing depth (4,926 versus 3,387 genes) and number of cells (18,590 versus 1,545 cells) led to a marked improvement in cell-type resolution (Supplementary Fig. 3a–f and Supplementary Tables 1 and 2). The classifier resolved the relation to Kathe et al.’s single-nucleus RNA-seq (snRNA-seq) data^29^ and confirmed that no neuron type was missing in the scRNA-seq dataset compared to the snRNA-seq dataset (Supplementary Fig. 4a–e).

**Extended Data Fig. 4:**
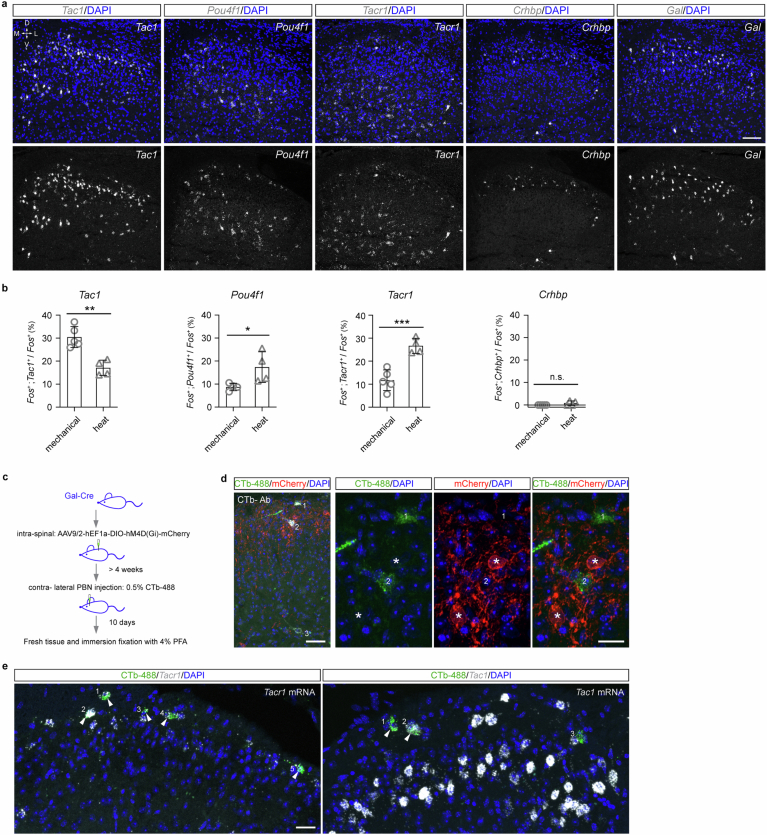
Expression of marker genes in mouse spinal dorsal horn, co-localization with *Fos* stimulated by mechanical or heat and retrograde labeling from lateral parabrachial nucleus. **a**, Representative images of *Tac1*, *Pou4f1*, *Tacr1*, *Crhbp* and *Gal* expression in mouse lumbar 4/5 spinal dorsal horn (n = 4 mice). DAPI was used as a counterstaining. Scale bar = 100 μm. M, medial; L, lateral; D, dorsal; V, ventral. **b**, Quantification for the percentage of *Fos*^+^ neurons that are *Tac1*, *Pou4f1*, *Tacr1* or *Crhbp* positive in spinal dorsal horn with mechanical or heat stimulation (n = 4 or 5 mice/group). Data are expressed as mean ± SD. Differences between the stimuli were analyzed by unpaired t test (two-tailed, *P* values in order 0.0018, 0.0454, 0.0008 and 0.0942). *** indicates *P* < 0.001, ** indicates *P* < 0.01, * indicates *P* < 0.05, and n.s. means no significance. **c**, Experimental paradigm for retrograde labeling of spinal *Gal*^+^ neurons from lateral parabrachial nucleus. **d**, Left: representative projection image (12 μm) of spinal dorsal horn showing non-overlapping between *Gal*^+^ neurons (mCherry) and retrogradely labeled spinal parabrachial (SPB) projection neurons (CTb-488/green and CTb antibody/white) indicated by numeric numbers. Right: high magnification of SPB projection neurons and *Gal*^+^ neurons indicated by asterisks. Scale bars: 100 μm for the left and 20 μm for the inset. **e**, Representative projection images of spinal dorsal horn showing overlapping between retrogradely labeled SPB projection neurons and *Tacr1*^+^ neurons (left, 8 μm projection) or *Tac1*^+^ neurons (right, 10 μm projection) as indicated by arrowheads. Scale bar: 25 μm. n = 4 mice for (**d**) and (**e**). Source data

**Extended Data Fig. 5:**
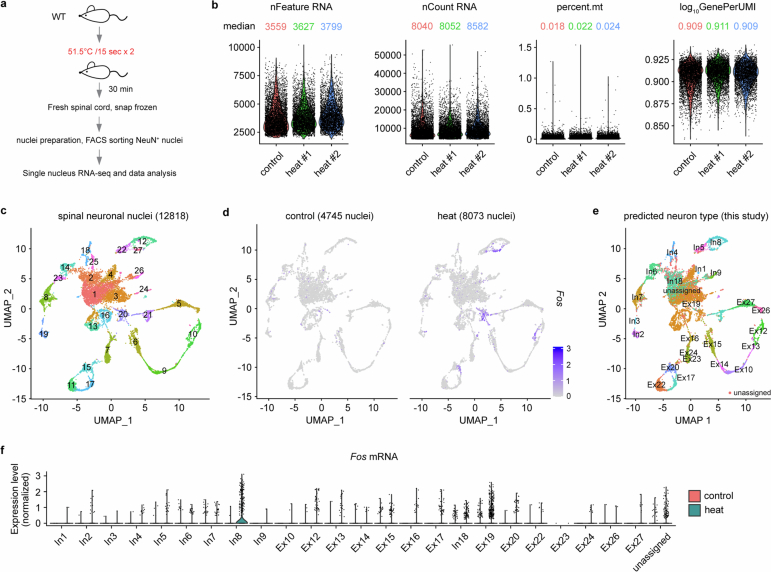
Single nucleus RNA sequencing of spinal cord from mice with noxious heat stimulation. **a**, Experimental paradigm for single nucleus RNA sequencing (snRNA-seq) of NeuN^+^ nuclei from mouse spinal cord after noxious heat stimulation. **b**, Violin plots showing neuronal nuclei passed quality control from control and heat samples. The medians were added on top of the plots. **c**, UMAP for spinal neuronal nuclei cell types from control and heat stimulated samples (12,818 nuclei). **d**, Feature plots for *Fos* expression in control (4,745 nuclei) and heat stimulated (8,073 nuclei) samples. Clusters 20 and 27 were unique in heat stimulated samples. **e**, UMAP for the neuronal nuclei dataset with predicted cell types defined in the current study with scPred. **f**, Violin plots showing *Fos* expression based on cell types between control and heat stimulated samples.

Next, neuron types from the mechanical and heat pain ensembles were identified by scRNA-seq. Ensembles were captured in Fos^dsTVA^*R26^Tom^ mice by intraspinal delivery of EnvA^M21^ lenti-Cre virus coincident with respective stimulation. Tomato^+^ cells were sorted, sequenced by scRNA-seq and mapped to the spinal cord atlas^30^ (Fig. 3b). Very few neurons from unstimulated mice were captured (Fig. 3b), while quantification of active neuron types in the different experimental conditions revealed the galanin (*Gal*) In8 neuron type as the only robustly activated inhibitory type, and this was a shared feature for mechanical and heat stimuli (Fig. 3c). By contrast, among the activated excitatory neurons, Ex21 and Ex19 neurons were dominant for mechanical and heat stimuli, respectively, as assessed by analysis with scCODA^31^ (Fig. 3c). Multiplexed RNA in situ hybridization of markers for these neuron types validated this finding (Fig. 3d), revealing coexpression of *Fos* and *Tac1* in mice subjected to a mechanical stimulus, whereas *Fos* and *Pou4f1* and *Fos* and *Tacr1* were coexpressed in mice subjected to a heat stimulus (Fig. 3e and Extended Data Fig. 4a,b). Although some deeper *Fos*^+^ neurons coexpressed these markers, most were laminae I/II neurons. Other neuron types (for example, *Crhbp*^+^ Ex25 neurons; Fig. 3e) showed either no contributions or proportionally smaller contributions to the ensembles. Spinoparabrachial (SPB) neurons that convey information related to mechanical and thermal noxious stimuli express *Tacr1*, *Gpr83* and/or *Tac1*, and some of the neurons expressing these markers display unique supraspinal target innervation, even though *Tacr1*/*Gpr83* and *Tacr1*/*Tac1* are coexpressed in several neuronal populations^10,11,17,32^. We confirmed that SPB neurons retrogradely labeled with cholera toxin subunit B (CTb) expressed *Tacr1* (95 ± 4%) or *Tac1* (33 ± 15%), but none expressed *Gal* (Extended Data Fig. 4c–e). *Tacr1*^+^ neurons are essential for responses to highly noxious stimuli and central sensitization^33,34^. Among the *Tacr1*-expressing neuron types (Ex13, Ex17, In18, Ex19 and Ex20), *Gpr83* was found to be expressed in Ex17 and Ex20 populations, whereas the expression of *Tac1* was detected at a low level in several populations (In2, In3, Ex11, Ex17, Ex19, Ex20, Ex21 and Ex22) with abundant expression in the Ex21 population (Fig. 3d). This is consistent with *Tacr1*^+^*Pou4f1*^+^*Gpr83*^−^ Ex19 SPB neurons encoding noxious heat and *Tac1*^+^ Ex21 SPB neurons encoding intense mechanical stimulation (Fig. 3b–d). Beyond stimulus-selective activation of spinal neurons, several other excitatory and a few inhibitory neurons represented shared neurons in the ensembles (Fig. 3c). snRNA-seq of spinal cords (12,818 neuronal nuclei) from mice stimulated with noxious heat and control mice confirmed Ex19 (*Tacr1*) and In8 (*Gal*) neurons as the main active populations based on stimulus-induced *Fos* expression (Extended Data Fig. 5a–f). Thus, ensembles encoding noxious mechanical and heat stimuli include separate neural representations and predict a single spinal inhibitory molecular neuron type uniquely expressing *Gal* to be recruited in a feed-forward manner to dampen excitatory responses regardless of modality.

### Ensemble transition in neuropathy

**Fig. 4:**
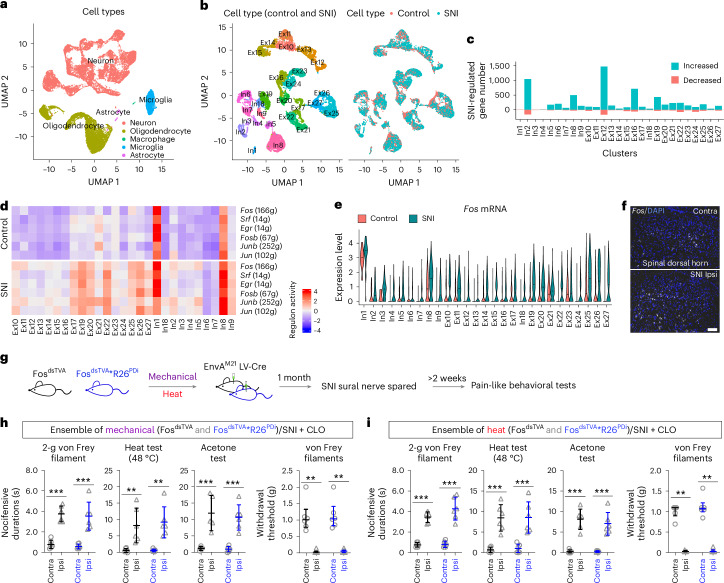
Spinal ensembles transition from nociceptive to chronic pain. **a**, UMAP composed of 28,195 cells sequenced by scRNA-seq (*n* = 4 control mice and *n* = 4 SNI mice; 6 weeks). **b**, UMAP showing neuronal cell types of 17,229 neurons (left) from both SNI (11,234) and control (5,995) conditions (right). **c**, Numbers of genes regulated by SNI (SNI versus control) for each cell type by pseudobulk analysis are listed. Genes with a log_2_(fold change) of >0.59 or <–1.0 and adjusted *P* value of <0.01 were counted as regulated by SNI. **d**, Heat map of immediate early genes (g) related regulon activities in neuronal cell types from control and SNI mice from SCENIC analysis. **e**, Violin plot showing *Fos* expression in control and SNI mice in all neuronal cell types. **f**, Representative image of *Fos* expression in the contralateral and ipsilateral spinal dorsal horn after SNI (*n* = 4 mice). DAPI was used as a counterstain; scale bar, 100 μm. **g**, Experimental paradigm for the role of mechanical and heat ensembles in neuropathic pain. **h**,**i**, Nocifensive durations for the 2-g von Frey filament test, heat test and acetone test and withdrawal thresholds for von Frey filaments were recorded to assess the effects of inhibition of mechanical and heat ensembles by clozapine after SNI (*n* = 24 mice, 6 mice per group). Data are expressed as mean ± s.d. or median with interquartile range (withdrawal threshold data for von Frey filaments). Differences between contralateral and ipsilateral sides within the group were analyzed by ordinary one-way ANOVA followed by a Bonferroni multiple comparisons test (adjusted *P* values from left: *P* < 0.0001, *P* < 0.0001, *P* *=* 0.0042, *P* *=* 0.0014, *P* *=* 0.0002 and *P* *=* 0.0002 in **h** and *P* < 0.0001, *P* < 0.0001, *P* *=* 0.0001, *P* *=* 0.0002, *P* < 0.0001 and *P* < 0.0001 in **i**). The differences in withdrawal threshold for von Frey filaments between contralateral and ipsilateral sides from each group were analyzed by Mann–Whitney test (two-tailed; *P* = 0.0022 for Fos^dsTVA^ and *P* = 0.0022 for Fos^dsTVA^*R26^PDi^ in **h** and *P* = 0.0022 for Fos^dsTVA^ and *P* *=* 0.0022 for Fos^dsTVA^*R26^PDi^ in **i**); ***P < 0.001 and **P < 0.01. Source data

**Extended Data Fig. 6:**
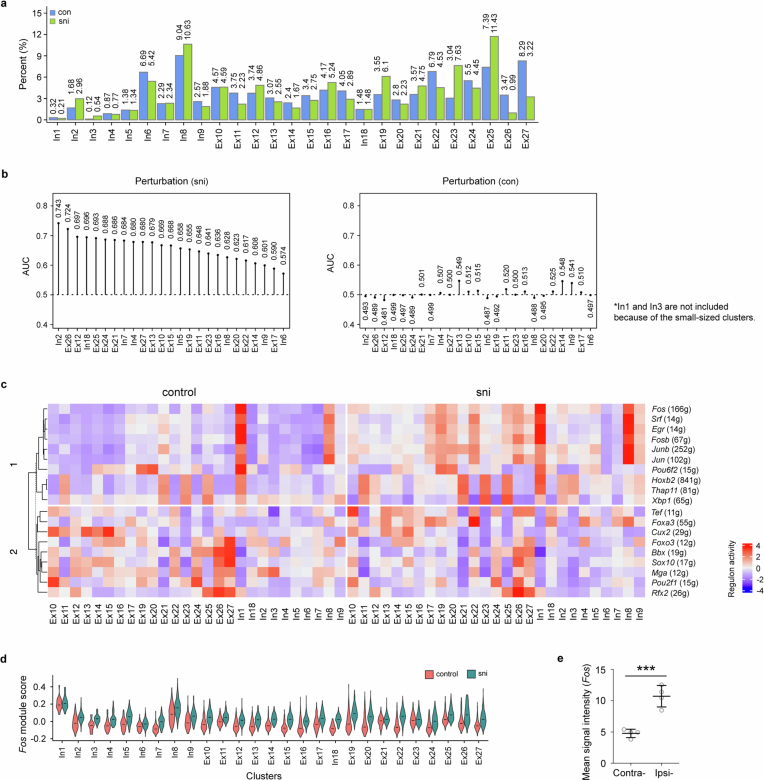
Effects of sni on neuronal cell types in mouse spinal dorsal horn. **a**, Effect of sni on the size (proportion) of neuronal cell types in mouse spinal dorsal horn. Con, control. **b**, Left: Perturbation analysis of sni on neuronal subtypes in spinal dorsal horn with Augur method. Right: Perturbation analysis on neuronal subtypes from a randomly selected control sample as negative control was also included. **c**, Enriched regulons for spinal dorsal horn neuronal cell types from control and sni mice by SCENIC analysis. **d**, Violin plot of module score for the list of genes from *Fos* regulon (166 g) in neuronal cell types from control and sni. **e**, Quantification of mean signal intensity for *Fos* mRNA (subtracted background, n = 4 mice). Data are expressed as mean ± SD. Differences between the stimuli were analyzed by unpaired t test (two-tailed, *P* = 0.0007). *** indicates *P* < 0.001. Source data

**Extended Data Fig. 7:**
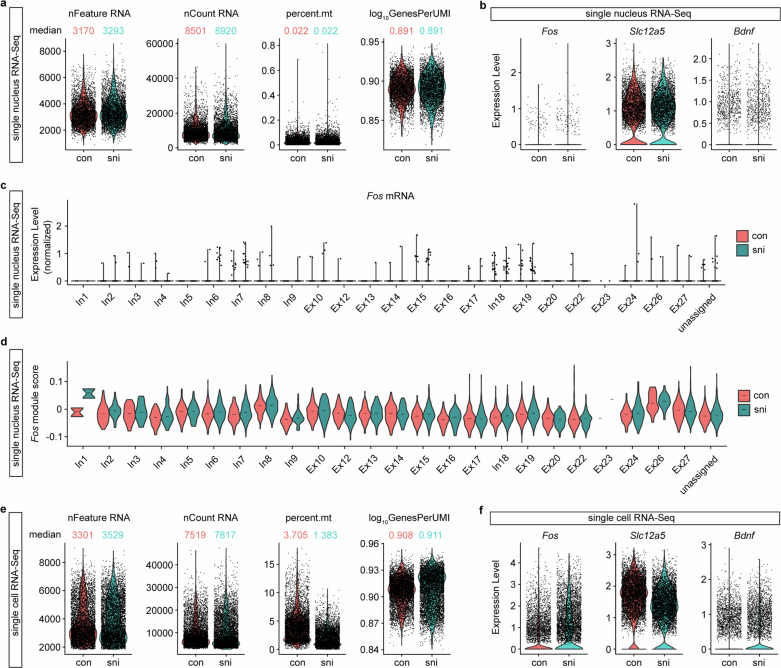
Effects of sni on neuronal cell types in mouse spinal dorsal horn from snRNA-seq. **a**, Violin plots showing neuronal nuclei passed quality control from control and sni samples (4,160 nuclei each). Only protein-coding genes were included. The medians were added on top of the plots. There were 12,428 nuclei from control samples passed quality control and down sampled to 4,160 nuclei for comparison with sni sample. **b**, Violin plots showing *Fos*, *Slc12a5* and *Bdnf* gene expression between con and sni samples. **c**, Violin plots showing *Fos* expression based on predicated cell types in the current study between con and sni samples. **d**, Violin plots showing *Fos* module score based on cell types between con and sni samples. **e**, Violin plots showing neuronal cells passed quality control from control and sni samples (5,995 cells each). The medians were added on top of the plots. Protein-coding genes (not Y chromosome) were included for the analysis here. **f**, Violin plots showing *Fos*, *Slc12a5* and *Bdnf* gene expression between con and sni samples from scRNA-seq.

**Extended Data Fig. 8:**
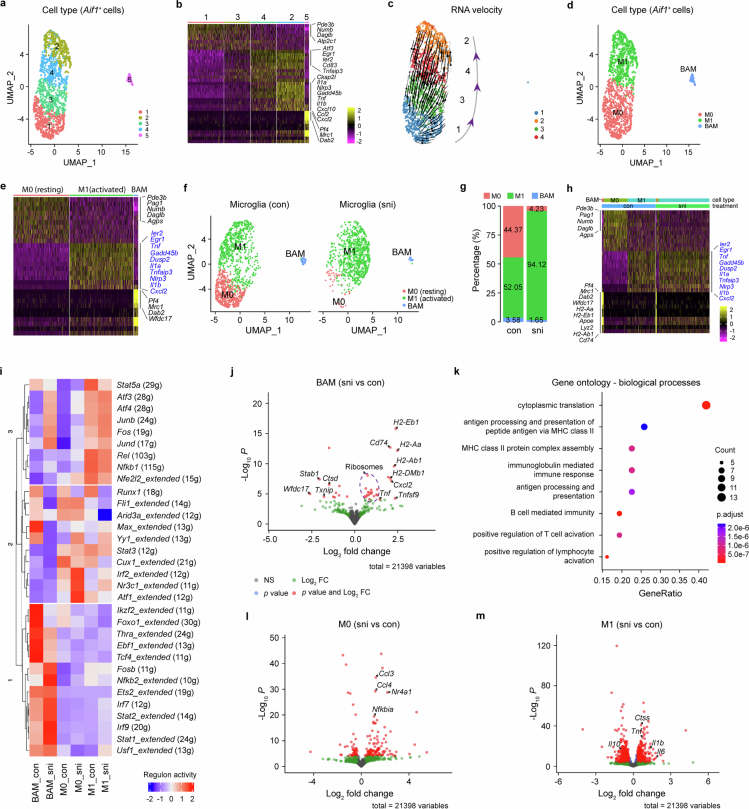
Effects of sni on *Aif1*^+^ cells in mouse spinal dorsal horn. **a**, UMAP plot shows 5 clusters of *Aif1*^+^ cells (1,368 cells) from spinal cord of control mice. **b**, Heatmap shows top 10 differently expressed genes (avg_logFC) for each cluster. **c**, RNA velocity shows microglia activation tracjectory. Although cells of cluster 5 are *Aif1*^+^, they are border associated macrophages (BAM) according to specifically expressed genes in (**b**). **d**, UMAP plot shows the annotation of *Aif1*^+^ glia cells (M0, resting microglia; M1, activated microglia) according to top differently expressed genes, RNA velocity analysis and literature. **e**, Heatmap shows top 10 differently expressed genes enriched based on the newly annotated glial subtypes (M0, M1 and BAM). **f**, UMAP plots show the composition of glial cells from control and sni mice. **g**, Stacked bar plot shows proportion of different glial cell types from control (1,368 cells) and sni (969 cells) mice. **h**, Heatmap shows top 10 differently expressed genes from BAM, M0 and M1 cell types under control and sni conditions. Only 950 glial cells from each sample were included for visualization by down sampling. **i**, Heatmap of regulon activity enriched for glial cell types. **j**, Volcano plot shows sni induced regulated genes BAMs. Dashed line circle indicates genes coding for ribosomal proteins. **k**, Gene ontology analysis revealed sni regulated genes in BAMs were mainly involved in protein translation and immune response processes. **l**, Volcano plot shows sni induced regulated genes in resting microglia (M0). **m**, Volcano plot shows sni induced regulated genes in activated microglia (M1). Pro-inflammatory cytokine genes *Ccl3*, *Ccl4*, *Il1b* and *Il6* or anti-inflammatory cytokine gene *Il10* were indicated with arrows in (**l** and **m**). Genes plotted in **j**-**m** were analyzed with DESeq2 with absolute log_2_FoldChange > 0.3 and *P* < 1.0×10^−5^ (colored in red).

**Extended Data Fig. 9:**
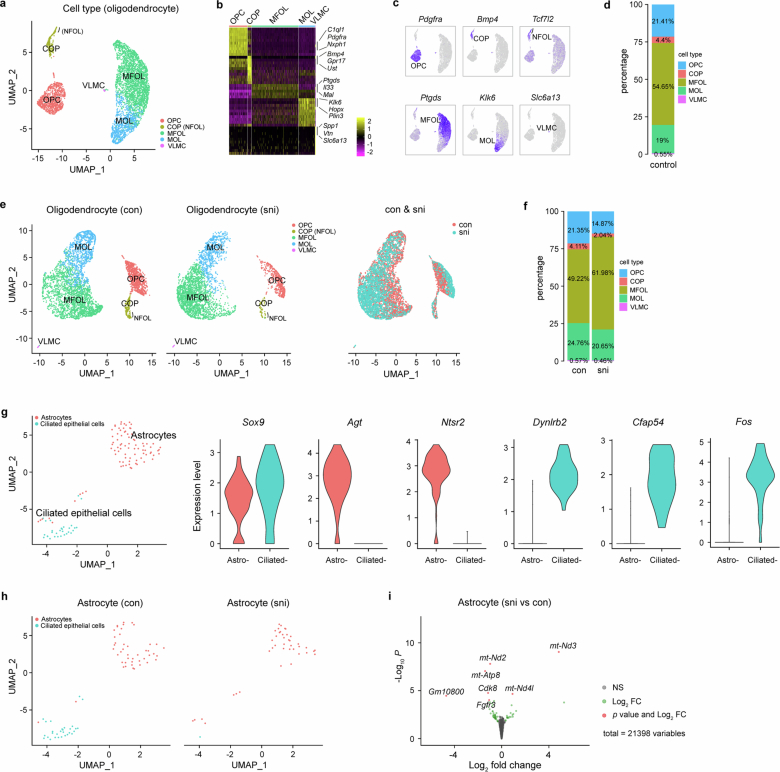
Effects of sni on oligodendrocytes and astrocytes in mouse spinal dorsal horn. **a**, UMAP plot shows different cell subtypes along oligodendrocyte lineage from mouse spinal cord based on selected markers of Marques’s 2016 annotation. OPC, oligodendrocyte precursor cells; COP, differentiation-committed oligodendrocyte precursors; NFOL, newly formed oligodendrocytes; MFOL, myelin-forming oligodendrocytes; MOL, mature oligodendrocytes. **b**, Heatmap shows top 10 differently expressed genes (avg_logFC) for different oligodendrocyte subtypes. **c**, Feature plots show selected marker genes for different oligodendrocyte subtypes. **d**, Stacked bar plot shows the population sizes of different oligodendrocyte subtypes. **e**, UMAP plots show oligodendrocytes subtypes from spinal cord of control mice (4,750 cells) and mice with sni (3,719 cells). **f**, Stacked bar plot shows the population sizes of different oligodendrocyte subtypes from con and sni conditions. **g**, Left: UMAP plot shows 2 clusters of *Sox9*^+^ cells (116 cells) from spinal cord of control and sni mice. Right: Violin plots show *Agt* and *Ntsr2* are specifically expressed in astrocytes, whereas *Dynlrb2* and *Cfap54* are limited to ciliated epithelial cells (ependymal cells). *Fos* is highly expressed in ciliated epithelial cells, like CSF contacting neurons (In1, Fig. 4e). **h**, UMAP plots show astrocytes and ciliated epithelial cells from spinal cord of control mice (74 cells) and mice with sni (32 cells). **i**, Volcano plot shows sni induced gene expression in astrocytes analyzed by DESeq2 with absolute log_2_FoldChange > 0.3 and *P* < 1.0 × 10^−5^ (colored in red).

To test whether the quality-selective neural ensembles represent unique ensembles for nociceptive pain or whether neuropathic pain induced by spared nerve injury (SNI) share these circuits, we performed scRNA-seq on spinal cord segments corresponding to the central arborizations of the sciatic nerve from mice subjected to SNI and control mice. In total, 28,195 cells passed quality control, of which 17,229 were neurons and the rest were border-associated macrophages (BAMs), microglia, oligodendrocytes and astrocytes (Fig. 4a). Neurons from both control and SNI mice clustered into the same cell types as in the original reference neuron atlas (Fig. 4b), with largely unaltered population size representation (Extended Data Fig. 6a). However, differential gene expression analysis showed that up to 1,000 genes were affected in most neuronal types (Fig. 4c and Supplementary Tables 3 and 4). Machine learning-based perturbation analysis^28^ further confirmed a marked effect on most neuronal cell types (Extended Data Fig. 6b). Computational reconstruction of the gene regulatory network (GRN) with single-cell regulatory network inference and clustering (SCENIC)^35^ uncovered broad effects on the activities of regulons (that is, transcriptional regulator and *cis*-regulatory target genes) for several activity-induced genes, whereas other regulons were largely unaffected. The activity-dependent regulons (*Fos*, *Srf*, *Egr*, *Fosb*, *Junb* and *Jun*) were robustly increased in SNI mice compared to in control animals in Ex10, E17, Ex19, Ex20, Ex22 and Ex25–E27 populations as well as in In8 and In9 neurons (Fig. 4d and Extended Data Fig. 6c). *Fos* expression and *Fos* regulon module score analyzed in the scRNA-seq data (Supplementary Table 5) were increased in most, if not all, of these neuron types, consistent with increased expression of *Fos* in ipsilateral spinal dorsal horn neurons compared to in neurons contralateral to the lesion analyzed by in situ hybridization (Fig. 4e,f and Extended Data Fig. 6d,e). We also performed snRNA-seq on 16,588 nuclei prepared from the spinal cords of mice 4 weeks after SNI and from control mice (Extended Data Fig. 7a). However, the expression of known nerve injury-regulated genes *Slc12a5* (encoding KCC2 and downregulated by peripheral nerve injury)^36^ and *Bdnf* (upregulated by injury)^37^ as well as *Fos* and *Fos* module score were not as robust as in the scRNA-seq data (Extended Data Fig. 7b–f). Thus, our results show a broad disinhibition of numerous excitatory neuron types, suggesting an altered environment (for example, inflammation). We next analyzed microglia, macrophages, oligodendrocytes and astrocytes (Extended Data Figs. 8 and 9). Cell clustering, differential gene expression and RNA velocity analyses in *Aif1*^*+*^ cells (microglia from control mice) showed microglia to mainly be resting (M0) or activated (M1, proinflammatory; Extended Data Fig. 8a–e). In the chronic phase after nerve injury, resting microglia were almost absent, with a proportional increase in activated microglia (Extended Data Fig. 8f–h). RNA velocity showed activated microglia arising from the resting subtype (Extended Data Fig. 8c). No subpopulation composition^38^ alterations were observed in oligodendrocytes (Extended Data Fig. 9a–f) or astrocytes (Extended Data Fig. 9g–i). Thus, microglia activation could therefore explain the marked perturbation of spinal neurons during the chronic stage of neuropathic pain, as has been proposed previously^39–41^. SCENIC analysis revealed increased regulon activity of NF-κB (*Nfkb2*) and type I interferon (*Irf7*, *Irf9* and *Stat1*) in macrophages and immediate early gene regulons in activated M1 microglia and macrophages after SNI compared to the resting state (Extended Data Fig. 8i). Differential gene expression and Gene Ontology analysis confirmed a notable increase in antigen presentation via major histocompatibility complex class II and signaling for lymphocyte activation in macrophages (Extended Data Fig. 8j,k), consistent with interferon-stimulated gene expression, and enrichment of proinflammatory cytokines such as *Ccl3* and *Ccl4* in resting (M0) microglia and *Tnf*, *Il1b* and *Il6* in activated (M1) microglia in animals with neuropathic pain (Extended Data Fig. 8l,m). These results indicate a spinal inflammatory reaction driven by BAMs and microglia during peripheral neuropathy. To examine the functional consequences of chronic pain, neural ensembles representing mechanical and heat pain were first captured for hM4Di-DREADD expression followed by SNI (Fig. 4g). Silencing the noxious mechanical and heat spinal nociceptive ensembles did not resolve nor relieve mechanical allodynia and hypersensitivity to blunt pricking (2-g von Frey filament) or heat or cold stimulation (Fig. 4h,i). These results indicate a transition of ensembles from acute nociceptive pain to chronic allodynia and hyperalgesia. The transition correlated with a predicted inflammatory environment and manifested in a disinhibition of numerous spinal excitatory neuron types.

### *Gal*^+^ neurons gate nociception and reverse neuropathic pain

**Fig. 5:**
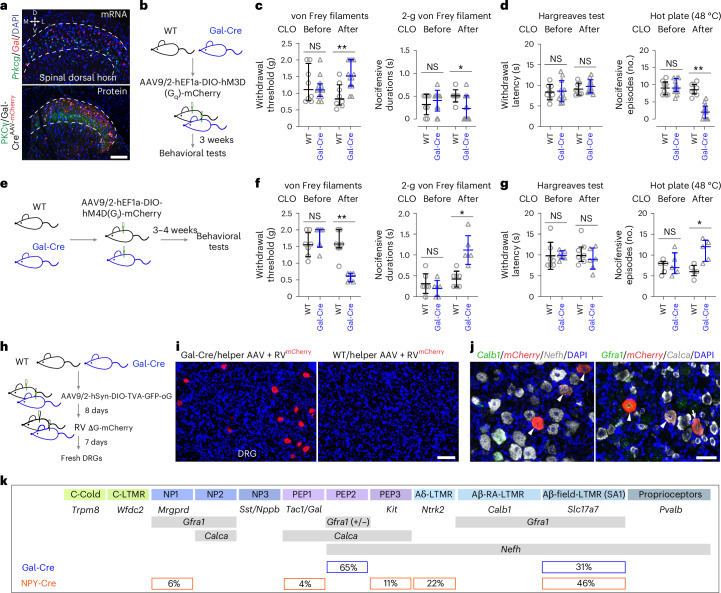
*Gal*^+^ In8 neurons modulate nociceptive pain. **a**, Top: *Gal* and *Prkcg* expression in the mouse dorsal horn. Bottom: mCherry and PKCγ expression in the spinal dorsal horn of Gal-Cre mice injected with AAV9/2-DIO-mCherry; *n* = 4 mice per experiment. **b**, Experimental paradigm for the gain-of-function study of *Gal*^+^ In8 neurons (**c** and **d**); WT, wild-type. **c**,**d**, von Frey filament and 2-g von Frey filament tests (**c**) as well as a Hargreaves test and hot plate test (**d**) were assessed; *n* = 8 wild-type and 12 Gal-Cre mice. **e**, Experimental paradigm for the loss-of-function study of *Gal*^+^ In8 neurons. **f**,**g**, The same tests were assessed as in **c** (**f**) and **d** (**g**); *n* = 7 wild-type and 6 Gal-Cre mice and 5 mice per group in the hot plate test. **h**, Experimental paradigm for monosynaptic retrograde tracing from spinal *Gal*^+^ neurons to DRG neurons. **i**, Retrograde-traced DRG neurons were labeled with mCherry in Gal-Cre and wild-type mice (*n* = 2 mice per group). **j**, Identification of traced mCherry^+^ DRG neurons with RNAscope probes (*n* = 2 mice). Arrow indicates triple labeling, and arrowheads indicate double labeling. **k**, Summary for identified traced neurons with DRG neuronal subtype marker genes in Gal-Cre and NPY-Cre mice. Some traced neurons could not be assigned with the probes used here. DAPI was used as a counterstain; scale bars, 100 μm (**a** and **i**) and 50 μm (**j**). Data are expressed as mean ± s.d., whereas nocifensive episodes and withdrawal threshold are expressed as median with interquartile range. Differences between wild-type and Gal-Cre mice in **c**, **d**, **f** and **g** and clozapine were analyzed by ordinary one-way ANOVA followed by a Bonferroni multiple comparisons test, whereas the nonparametric data were analyzed by Kruskal–Wallis test and followed by a Dunn’s multiple comparison test. Adjusted *P* values from left: *P* > 0.9999, *P* *=* 0.0069, *P* *=* 0.8541 and *P* *=* 0.0292 (**c**); *P* > 0.9999, *P* *=* 0.9555, *P* > 0.9999 and *P* *=* 0.0011 (**d**); *P* *=* 0.6666, *P* *=* 0.0085, *P* > 0.9999 and *P* *=* 0.0304 (**f**); and *P* *=* 0.9925, *P* *=* 0.4638, *P* > 0.9999 and *P* *=* 0.0177 (**g**); **P < 0.01 and *P < 0.05; NS, not significant. Source data

**Extended Data Fig. 10:**
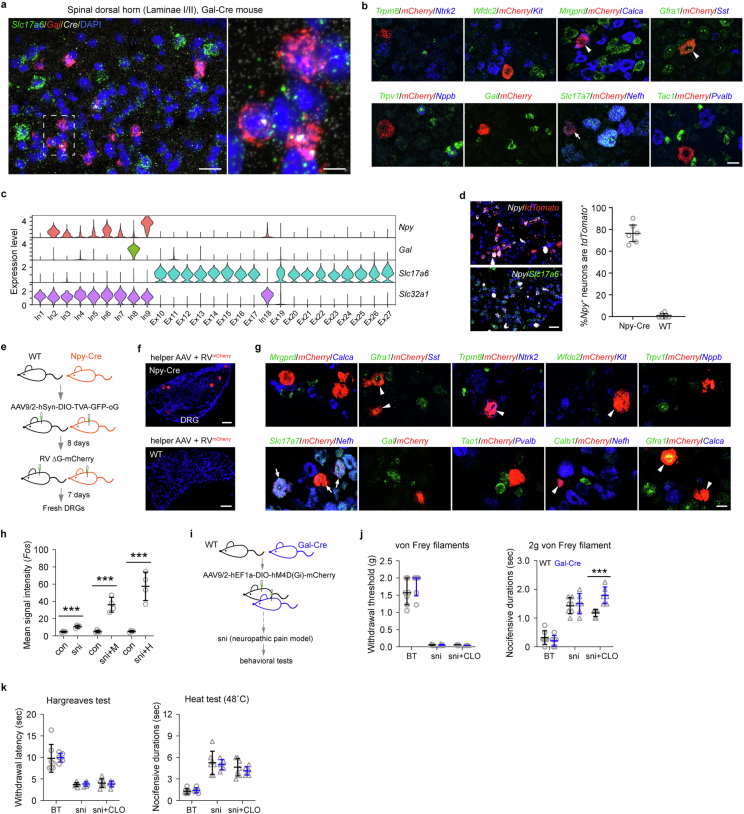
Identification of retrograde traced DRG neurons projecting to spinal *Gal*^+^ and *Npy*^+^ neurons. **a**, Representative image for *Slc17a6*, *Gal* and *Cre* in spinal dorsal horn from Gal-Cre mice (n = 3). High magnification images show co-localization of *Gal* and *Cre* from the inset. The asterisk indicated a triple labeled neuron. **b**, Identification of traced mCherry^+^ DRG neurons with genes covering DRG neuronal subtypes. **c**, Violin plots showing *Npy* and *Gal* expression in spinal cord. **d**, Validation of Npy-Cre*R26^Tom^ mice in spinal cord with RNAscope. The percentage of *Npy*^+^ neurons that were *tdTomato*^+^ was 77 ± 7.7% for Npy-Cre mice and 1.1 ± 1.6% for WT mice (n = 2 mice/group). *Npy*^+^ neurons barely co-localized with *Slc17a6* (0.5 ± 0.8%). The plotted dots stand for tissue sections. **e**, Experimental paradigm for monosynaptic tracing of spinal *Npy*^+^ neurons from DRGs. **f**, Traced DRG neurons were labelled with mCherry in Npy-Cre mice, but not in WT mice (n = 2 mice/group). **g**, Identification of traced mCherry^+^ DRG neurons as summarized in Fig. 5k. **h**, Quantification of *Fos* mRNA in spinal dorsal horn (n = 4 mice/group). con: contralateral, sni+M: sni with mechanical stimulation, sni+H: sni with heat stimulation. The sni data was also presented in Extended Data Fig. 6e. **i**, Experimental paradigm for chemogenetic silencing of *Gal*^+^ neurons in Gal-Cre mice with sni. **j**, Behavioral tests of von-Frey filaments and 2g von-Frey filament on sni mice with silenced spinal *Gal*^+^ neurons. **k**, Behavioral tests of Hargreaves and heat on sni mice with silenced spinal *Gal*^+^ neurons. n = 7 for WT and n = 6 for Gal-Cre mice in (**j** and **k**). Arrows indicate triple labelling and arrowheads indicate double labelling. Scale bars = 20 μm in (**a**), 5 μm for the inset from (**a**), 25 μm in (**b**, **d**, **g**) and 100 μm in (**f**). Data are expressed as mean ± SD, while withdrawal threshold is expressed as median with interquartile range. *Fos* intensity was analyzed by unpaired t test (two-tailed, *P* values from left 0.0007, 0.0004 and 0.0007. Differences for clozapine under sni condition between WT and Gal-Cre mice were analyzed by ordinary one-way ANOVA followed by Bonferroni’s multiple comparisons test, and withdrawal threshold was analyzed by Kruskal-Wallis test and followed by Dunn’s multiple comparison test. Adjusted *P* values from left > 0.9999, > 0.9999 and 0.2822 for von Frey filaments test, > 0.9999, > 0.9999 and 0.0005 for 2g von Frey filament test in (**j**), Adjusted *P* > 0.9999 for Hargreaves and heat tests between WT and Gal-Cre mice at BT, after sni and sni + CLO (**k**). *** indicates *P* < 0.001. Source data

GABAergic interneurons are effectors of inhibitory modulation of primary afferent signaling. Loss of inhibitory input in the spinal dorsal horn contributes to pain under physiological and pathological conditions^14,18,42,43^. *Gal*^+^ In8 neurons represent the major inhibitory neuron type in mechanical and heat ensembles. We examined the impact of these neurons on nociception and neuropathic pain. *Gal* expression in wild-type mice and Cre-dependent Tomato expression in Gal-Cre mice occurred mainly in neurons superficial to/intermingled with neurons expressing *Prkcg* (PKCγ) in laminae I and II (Fig. 5a), consistent with prior spatial transcriptomics data (GABA2/GABA3 in Häring’s annotation)^22^. The Gal-Cre driver mice faithfully recapitulated endogenous *Gal* expression (Extended Data Fig. 10a). Retrograde tracing from the lateral parabrachial nucleus in Gal-Cre mice confirmed *Gal*^+^ neurons as local interneurons (Extended Data Fig. 4c,d), consistent with previous findings^44^. Clozapine activation of Gal-Cre mice with intraspinal delivery of hM3D(G_q_) adeno-associated virus (AAV; Fig. 5b) had no effect on heat withdrawal latency but increased mechanical withdrawal threshold and decreased nocifensive responses to both noxious mechanical and heat stimulations (Fig. 5c,d). The relative influence of Gal-Cre neurons on nocifensive behavior was greater for the more intense noxious hot plate than for blunt pricking (2-g von Frey filament). Silencing of *Gal*^+^ neurons in Gal-Cre mice using hM4D(G_i_) led to a decreased mechanical withdrawal threshold and elevated nocifensive responses to both noxious mechanical and heat stimuli (Fig. 5e–g). Together, these data suggest that *Gal*^+^ neurons are recruited with increasing noxious intensity regardless of stimulus modality.

We next sought to identify the afferent input to Gal-Cre neurons. Retrograde rabies tracing from spinal *Gal*^+^ neurons with AAV^TVA^ helper virus in Gal-Cre mice revealed monosynaptic inputs from a group of dorsal root ganglia (DRG) neurons of medium (44%, 300–700 μm^2^) to large size (56%, >700 μm^2^; Fig. 5h,i). Markers for sensory neurons^45–47^ (Fig. 5j,k and Extended Data Fig. 10b) revealed a lack of traced unmyelinated nociceptive, thermoreceptive and pruriceptive types. Most were myelinated (*Nefh*^+^, 96%), and 38% were *Calca*^+^*Gfra1*^+^, 27% were *Calca*^+^*Gfra1*^−^, and 31% were *Calca*^*–*^*Gfra1*^+^ (Fig. 5j,k and Extended Data Fig. 10b). *Calca*^+^*Gfra1*^+/−^ (*Nefh*^+^) neurons were classified as PEP2 neurons and were thus presumed to be A-heat and high-threshold mechanoreceptive neurons^46,47^. *Calca*^*−*^*Gfra1*^+^ neurons were consistent with Aβ-field-low threshold mechanoreceptors (LTMRs) as they were negative for *Calb1* (marking Aβ-RA-LTMRs) and *Ntrk2* (marking Aδ-LTMRs), but *Slc17a7*^+^ neurons were exclusively expressed in touch-sensitive Aβ-LTMRs. Combined, this suggests a monosynaptic input to Gal*-*Cre neurons from PEP2 nociceptors and Aβ-field-LTMRs. Retrograde rabies tracing from spinal *Npy*^+^ inhibitory interneurons in NPY-Cre mice revealed monosynaptic inputs from Aβ-field-LTMR, Aδ-LTMR and PEP3 DRG neurons (Fig. 5k and Extended Data Fig. 10c–g).

**Fig. 6:**
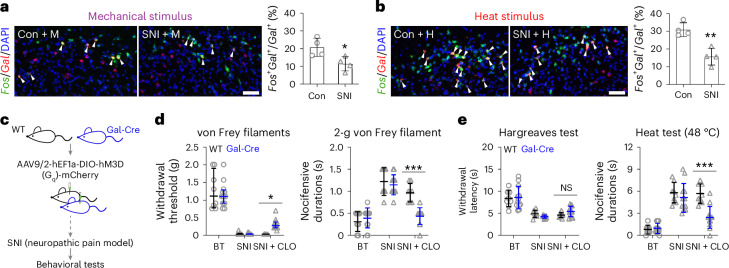
Role of *Gal*^+^ In8 neurons in neuropathic pain. **a**,**b**, Colocalization of *Fos*^+^ and *Gal*^+^ neurons in the spinal dorsal horn after mechanical (M; *n* = 4 mice per group; **a**) or heat stimulation (H; *n* = 4 mice per group; **b**). Arrowheads indicate neurons showing colocalization of *Fos* and *Gal*. Right: quantification for the percentage of *Gal*^+^ neurons that were *Fos*^+^ is plotted. Differences between control and SNI mice with stimulation were analyzed by unpaired, two-tailed *t*-test (*P* = 0.0291 in **a** and *P* = 0.0026 in **b**). **c**, Experimental paradigm for the effect of gain of function for *Gal*^+^ In8 neurons in SNI-induced chronic pain. **d**, Withdrawal threshold for mechanical and nocifensive durations for noxious mechanical tests on SNI mice with activation of *Gal*^+^ neurons. **e**, Withdrawal threshold for Hargreaves and nocifensive durations for noxious heat tests on SNI mice with activation of *Gal*^+^ neurons. These SNI mice were the same as in Fig. 5b–d, and the basal thresholds (BT) were the same values as those before clozapine treatment for the same tests. DAPI was used as a counterstain (**a** and **b**); scale bars, 50 μm. Data are expressed as mean ± s.d., whereas withdrawal threshold is expressed as median with interquartile range. Differences in the effects of clozapine in SNI mice between wild-type and Gal-Cre mice (**d** and **e**) were analyzed by ordinary one-way ANOVA followed by a Bonferroni multiple comparisons test, whereas the withdrawal threshold was analyzed by Kruskal–Wallis test followed by a Dunn’s multiple comparison test. Adjusted *P* values from left: *P* > 0.9999, *P* > 0.9999 and *P* *=* 0.0418 for the von Frey filaments test and *P* > 0.9999, *P* > 0.9999 and *P* < 0.0001 for the 2-g von Frey filament test in **d** and *P* > 0.9999, *P* > 0.9999 and *P* *=* 0.6952 for the Hargreaves test and *P* > 0.9999, *P* *=* 0.8136 and *P* < 0.0001 for the heat test in **e**; ***P < 0.001, **P < 0.01 and *P < 0.05. Source data

As mentioned previously, an overall increase in *Fos*, *Fos* module score and activity-dependent regulons (Fig. 4e,f and Extended Data Fig. 6d,e) indicated an overall disinhibition of neurons. However, these SNI-induced changes were modest compared to stimulus-induced *Fos* expression (Extended Data Fig. 10h). It therefore remained possible despite overall increased spinal activity that an imbalance of the excitatory/inhibitory drive contributes to hyperalgesia. Control and SNI mice (18 days) received either a mechanical or heat stimulus and were killed 30 min later. Quantification of the percentage of *Gal*^+^ neurons that were *Fos*^+^ in the spinal dorsal horn revealed a reduction of mechanical and heat recruitment of *Gal*^+^ neurons in mice with SNI compared to control mice (Fig. 6a,b). Thus, *Gal*^+^ neurons can contribute to reduced spinal inhibitory tone, and, hence, restoring activity could reverse neuropathic pain. SNI performed on mice with hM3D(G_q_) expression in Gal-Cre spinal neurons without clozapine (Fig. 6c) led to increased nocifensive behavior and reduced withdrawal thresholds compared to prior SNI (Fig. 6d,e). Clozapine activation of Gal-Cre neurons in these mice reversed neuropathy-induced nocifensive behavior to mechanical and heat stimuli, with a smaller effect or no effect on withdrawal thresholds, respectively (Fig. 6d,e). Silencing Gal-Cre neurons in SNI mice further increased responses to noxious mechanical stimuli, but not noxious heat stimuli or thresholds to mechanical and heat stimuli (Extended Data Fig. 10i–k). The essential role of In8 neurons in the mouse made us examine whether this applies for cross-species homologous neurons in the human spinal cord. Correlation analysis of GRNs between the mouse and human spinal cord^48^ datasets revealed conserved *Gal*^+^ In8 populations in the human spinal cord (Inh-Dorsal-8 and Inh-Dorsal-6; Supplementary Fig. 5), suggesting that a similar gating mechanism could also exist in humans.

## Discussion

Here, we examine the neural basis for one of the most fundamental mechanisms enabling protection from harmful stimuli by using several recent technological advances, including the high-temporal-resolution CANE approach for genetic capture of active neurons, scRNA-seq, machine learning classifiers and functional manipulation of discreet ensembles and neuron types. We discover that neural activity alone within spinal ensembles is sufficient to recreate and completely abolish the sense of mechanical- and heat-induced pain. Thus, the identified ensembles are the carriers of cutaneous noxious mechanical and heat stimuli. Although primary sensory neuron input involves several neuron types^46^, the simplicity of spinal ensembles indicates these to encode the input into discreet and dedicated circuits with only a few molecular neuron types responsible for several dimensions experienced by noxious external stimuli. Our results suggest that the ensemble cell-type composition represents stimulus modality, valence and intensity because subthreshold ensemble activation potentiated pain-related behavior. Intensity could be scaled by increasing active neuron numbers and/or firing frequency of already active neurons, as has previously been proposed^49^. Although forced activation impacts the withdrawal reflex, silencing the ensembles had no effect. It is unclear why ensemble inhibition is without effect. A possible reason could be a nonproportional increase of *Fos* expression and therefore captured neurons involved in nocifensive responses compared to those involved in withdrawal. However, if the same ensembles encode nocifensive and withdrawal responses but much fewer neurons need to be recruited for initiating a withdrawal response, inhibition experiments could be unsuccessful while activation would suffice. Although we cannot exclude that the identified neurons in the ensembles are also necessary for withdrawal reflexes, recent studies suggest unique spinal neurons that encode the withdrawal reflex versus nocifensive-related behavior, as defined here^10,50^.

Our results show that mechanical and heat stimuli on the skin are represented by spinal ensembles that contain a weaker recruitment of excitatory neurons shared between stimuli and stronger recruitment of stimulus-unique neuron types. Among the modality-selective activated excitatory neurons, Ex21 neurons, defined by *Tac1* expression, encode intense noxious mechanical stimuli, and Ex19 neurons, defined by *Pou4f1*/*Tacr1* expression, represent intense heat. Spinal *Erbb4*^+^ interneurons have been recently shown to participate in noxious heat sensation associated with protective reflexive responses, which governs heat sensation together with *Sst*^+^ and *Cck*^+^ neurons^9^. However, *Erbb4* is widely expressed in the spinal cord, such as in In5, In9, Ex12–Ex15, Ex17–Ex19 and Ex27 neurons, which cover all excitatory neuronal populations together with *Sst* and *Cck* in the current neuronal atlas. Mice lacking spinal *Tac1*^*+*^ neurons display a loss of the nocifensive behavior associated with noxious stimuli, such as aversion and licking, but not protective reflexive responses^10^. This phenotype was observed after both noxious mechanical and heat stimuli, suggesting that *Tac1*^+^ neurons have a general role in driving the nocifensive component of sustained pain^10^. Our finding that the mechanical and heat ensembles mainly control nocifensive behavior is consistent with the ablation of *Tac1*-expressing spinal neuronal types. However, *Tac1* is also expressed at lower levels in other populations of the dorsal spinal cord^22,24^, consistent with our new atlas data, that is, Ex17 and Ex19–Ex22. We found that when reactivating ensembles, responses were facilitated without any cross-modality spillover. Thus, even with artificially forced activity, the ensembles stay tuned. This shows that the ensembles represent discrete processing units required and sufficient by themselves to encode noxious mechanical and heat stimuli.

Inhibitory neurons of the spinal dorsal horn exert critical control over the relay of mechanical and heat nociceptive signals to higher brain areas^14^. Spinal excitatory neuron types converge with feed-forward inhibition, and the interdependence of the spinal network components together shapes the spinal output signal, which carries information about environmental experiences conveyed by primary sensory neurons^5,45^. We interpret In8 neurons as feed-forward inhibitory neurons that gate excitatory output from heat and mechanical ensembles because enhancing the activity of *Gal*^+^ In8 neurons ameliorated pain-like behavior in response to both mechanical and heat stimuli. The lack of cross-ensemble effects indicates that the mechanical and heat ensembles, which both include In8 neurons, are organized functional units, as has been shown for sensorimotor reflexes^50,51^. Our results are in contrast to previously studied parvalbumin-expressing (*Pvalb*; expressed in In3, In4, Ex14 and Ex15 neurons) and prodynorphin-expressing (*Pdyn*, expressed in In5 and In8 neurons) dorsal horn interneurons, which have been shown to be involved in circuit-gating protective reflexes by A-LTMR^8,18^ and directly inhibit SPB neurons. Inhibitory neurons expressing potassium voltage-gated channel interacting protein 2 (*Kcnip2*) are preferentially activated by cold but unlike *Gal*^+^ neurons have no impact on noxious mechanical or heat pain^52^. The relation of function to other inhibitory interneurons previously studied functionally, such as those expressing nitric oxide synthase 1 (*Nos1*)^53^ and NPY, is difficult to assess as these markers are expressed in multiple excitatory and/or inhibitory neuron types. Thus, unlike these interneurons, *Gal*^+^ In8 neurons represent a key inhibitory neuron type for defensive nocifensive behavior elicited by noxious stimuli.

Our findings of direct monosynaptic connectivity with nociceptors are consistent with In8 neurons as feed-forward inhibitory neurons in the pain pathway. Thus, spinal neurons activated by noxious stimuli are likely to primarily function to transmit pain (pro-pain), but antinociceptive *Gal*^+^ inhibitory neurons appear to be recruited in a feed-forward manner to dampen excitatory responses. Feed-forward inhibitory neurons in the spinal sensorimotor pathway are essential in the processing of touch stimuli and shape the representation of touch in the somatosensory cortex^12,54^. Similarly, it is conceivable that In8 neuron feed-forward inhibition improves acuity and contrast through lateral inhibition. Processing of pain-related information in the spinal cord can be modulated by descending modulatory pathways. One such pathway is the rostral ventromedial medulla (RVM) pathway, where excitatory antinociceptive RVM^BDNF^ neurons are monosynaptically connected with spinal *Gal*-expressing neurons^55^. Thus, based on the impact of *Gal*-expressing neurons on output spinal neurons and pain-related behaviors, it seems that feed-forward *Gal*^+^ In8 neurons also integrate supraspinal input onto the ascending pain pathway.

Nociceptive neurons can be categorized in terms of the preferred stimulus, threshold of activation and their conduction velocity. Heat sensation involves a range of cold, warmth and heat-detecting skin receptors, with a dominance of slow C-fiber over fast A-fiber heat nociceptors^46^. Similarly, high-force mechanical stimuli result in the activation of a range of both touch and nociceptive neuron types, which can be heat sensitive or insensitive, some of which are C-fibers and others A-fibers (A-high-threshold mechanoreceptors)^56,57^. In the current scRNA-seq^45,46^ taxonomy of mouse nociceptors and pruriceptors^46^, several molecularly defined C-fiber types have been shown to respond to heat or mechanical stimuli, while there is one predicted A-heat and A-high-threshold mechanoreceptor neuron type, named PEP2 (refs. ^46,47^). *Gal*^+^ In8 neurons receive monosynaptic inputs from PEP2 nociceptors. *Gal*^+^ neurons also connect to Aβ-field-LTMRs that form large receptive fields terminating as circumferential endings around hair follicles with sensitivity to gentle skin stroking^58^. Although C-fiber nociceptors are evolutionary conflated in humans compared to in mice (two versus four types), A-fiber nociceptors have expanded and include four molecular types in humans, whereas there are only two types in mice^59^. Thus, it is possible that feed-forward inhibition of fast nociception might play a more important role in humans than in rodents.

Hyperalgesia caused by peripheral neuropathy involves a range of spinal excitatory neurons that do not normally convey modality-selective mechanical and heat nociception. We draw this conclusion from analyses of active neurons during SNI, the marked circuit-wide molecular perturbation, the failure of nociceptive ensembles to functionally respect noxious information quality and the failure of nociceptive ensemble silencing to resolve allodynia and hyperalgesia in mice. Neuropathic pain resulting from peripheral nerve injury leads to colony-stimulating factor 1 (*Csf1*) release from primary afferents that drives microglia activation and secondary neuroinflammation in the spinal cord^40^. Microglia have an important role in initiating and maintaining pain and inflammation^39,60–63^. Our results concur with these studies as we found an increase in inflammatory cytokine expression in M1 microglia (including *Il1b*, *Tnf* and *Il6*) and downregulation of anti-inflammatory cytokine expression (*Il10*). Proinflammatory mediators are believed to enhance synaptic transmission to produce central sensitization and neuropathic pain^64,65^. The lack of GRNs associated with inflammation in neurons does not exclude the existence of post-transcriptional alterations downstream of receptors activated by inflammatory mediators but suggests that conventional inflammatory signaling pathways known to engage transcription are not responsible for gene expression alterations in spinal neurons. Instead, we found activity-regulated gene transcription across many kinds of excitatory neurons. Activity-dependent gene transcription included genes whose products are essential for synapse plasticity and long-lasting changes at synapses^66^. Such gene expression changes beyond microglial neuroinflammation could contribute to maintenance of hyperactive spinal circuits in the SNI neuropathy model.

Several different kinds of inhibitory spinal cord dorsal horn neurons have been implicated in sensitization and mechanical allodynia in neuropathy, including those that express prodynorphin^8^ and parvalbumin^18^. However, it is important to note that these markers are not exclusive to a single discreet inhibitory interneuron type, and several are also expressed in excitatory neurons, as shown by neurochemical studies^67^ and scRNA-seq efforts, such as our current study. Silencing the mixture of pro- and antinociceptive neurons, including *Gal*^+^ In8 neurons of nociceptive ensembles, was ineffective at reducing pain-related behaviors associated with SNI-induced hyperalgesia. By contrast, forced activation of spinal Gal-Cre neurons reversed mechanical- and heat pain-related behaviors, and silencing spinal Gal-Cre neurons enhanced pain-related behaviors. Unlike prodynorphin and parvalbumin neurons, Gal-Cre neurons only marginally affected mechanical allodynia (and heat-induced protective reflexes). The failure of intense mechanical or heat stimulation to fully activate *Gal*^+^ neurons in SNI mice compared to control mice suggests that an In8 neuron-dependent break in the excitation/inhibition balance in SNI drives a disinhibition of excitatory neurons. Although galanin receptors *Galr2* and *Galr3* are not expressed in spinal neurons, we find that the inhibitory *Galr1* receptor is conspicuously expressed specifically in Ex21, Ex23 and Ex24 populations. Galanin (GAL) administered intrathecally attenuates neuropathic pain behavior through GALR1 in rats^68^; hence, both GAL and GABA released from In8 neurons could yield excitatory/inhibitory balance.

Comprehensive strategies for pain relief across pain types are urgently needed. Our results provide a cellular basis for how the nocifensive dimension of cutaneous noxious mechanical and heat stimuli is encoded in the spinal cord. This could be helpful for the development of therapeutic strategies targeting pain while preserving sensory processes for the detection of noxious stimuli and for protective reflexes.

## Methods

### Animals

Wild-type female and male C57BL/6 mice (adult, ~12 weeks of age) were obtained from Charles River (Scanbur). Knock-in mice and transgenic strains used in this study are listed in Supplementary Table 6. All mice were house under standard conditions (20–22 °C and ~50% humidity) on a 12-h light/12-h dark cycle with free access to food and water, and experiments were conducted in accordance with Swedish policy for the use of research animals and approved by a local ethical committee (Stockholms Norra djurföröksetiska nämnd and Uppsala djurförsöksetiska nämnd). Animals were assigned randomly for behavioral experiments. No statistical methods were used to predetermine animal group sizes, but our group sizes were similar to those in previous reports^22,69^.

### Viruses

Lenti-Cre virus pseudotyped with mutated EnvA^M21^ was produced by the viral vector core at Duke University based on the protocol from Sakurai et al.^25^. AAVs were produced by the viral vector facility at the University of Zürich and Charité University, where EnvA RVΔG-mCherry^70^ was acquired. All information on the viruses used is listed in Supplementary Table 6.

### Peripheral sensory stimulation

Heat or mechanical stimulation was performed under deep anesthesia with isoflurane (Baxter). For heat stimulation, the left hind paw was dipped into a water bath (51.0–51.5 °C) for 15 s twice with an interval of 1 min. For mechanical stimulation, mechanical force (600 g) was applied using a Randall Selitto Paw Pressure Meter (World Precision Instruments) for 10 s and was repeated five to ten times with an interval of 1 min. Fos activation was visualized at the mRNA (fresh tissue, 30 min after stimulation) and protein levels (perfused tissue, 90 min after stimulation). Intraspinal injections of EnvA^M21^ lenti-Cre virus were applied to capture activated ensembles after sensory stimulation.

### Surgery

#### Intraspinal injections

Surgical procedures were performed under anesthesia with isoflurane^14^ after 30 min of peripheral sensory stimulation. Briefly, an incision was made on the back skin to expose the T13 (thoracic) and L2 (lumbar) vertebrae. The mouse was then mounted to a motorized stereotaxic frame (Stoelting and Neurostar), and the vertebral column (L1) was fixed with spinal adaptors (Harvard Apparatus). The muscles, spinous process and left vertebral lamina were removed to expose one side of the L5 segment. The dura in the middle position of the L5 segment was pierced about 400 µm left to the middle posterior spinal vein with a bevelled 30-gauge needle (BD Microlance). Viral vectors were injected at a depth of 300 µm with a pulled glass micropipette mounted on a 10-µl Hamilton syringe with an injection speed of 30 nl min^–1^ (300 nl; Pump 11, Harvard Apparatus). The glass micropipette was kept in place for 5 min after injection. Another two injections were made rostral and caudal in both directions about 1.2 mm away from the first injection site. The skin was closed with 5-0 silk stitches (Ethicon, Agnthos). Xylocaine and carprofen (Apoteket) were used to relieve pain caused by surgery. For monosynaptic retrograde tracing, a slightly modified intraspinal injection protocol was used. An incision was made through the skin to expose the gap between the T13 and L1 vertebrae. The spinal column was fixed with a clamp (NARISHIGE) through the stabilization of the L1 transverse process. The posterior longitudinal ligament and ligament flavum connecting T13 to L1 were separated to expose the spinal cord. Helper AAV (500 nl) was injected at 400 µm right to the midline at a depth of 400 µm on one side (one injection), and pseudotyped rabies virus (500 nl) was injected 8 days after the injection of helper virus. Mice were maintained for another 7 days. Subcutaneous injections of bupivacaine and carprofen before surgery and buprenorphine and carprofen after surgery were performed to reduce pain.

#### SNI

The skin of the mid-thigh from the left lateral surface was incised under anesthesia with isolfurane, and a separation was made directly through the biceps femoris muscle, exposing three terminal branches of the sciatic nerve^71^. The common peroneal and tibial nerves were tightly ligated and transected distally to the ligation, and a 1- to 2-mm piece of the nerve was removed from the distal stump. The muscle and skin were closed in two layers with 5-0 silk stitches. Xylocaine and carprofen were used to relieve pain caused by surgery.

#### Lateral parabrachial nucleus injection

Mouse heads were mounted on a motorized stereotaxic frame (Stoelting and Neurostar) under anesthesia with isoflurane. An incision was made through the midline of the skull, and the following coordinates were used for the lateral parabrachial nucleus: anterior–posterior –5.11 mm, medial–lateral 1.25 mm and dorsal–ventral –3.25 mm from the surface of the skull. A 10-min injection (30 nl min^–1^, 300 nl) of 0.5% Alexa Fluor 488-labeled CTb (CTb-488) was controlled by an Elite nanomite pump. The glass micropipette was kept in place for 5 min after injection. The skin was closed with 5-0 silk stitches. Xylocaine and carprofen were used to relieve pain caused by surgery, and mice were maintained for an additional 10 days.

### Drugs

Clozapine (1 mg ml^–1^ in ethanol as a stock solution) was delivered via intraperitoneal injection (0.2 mg per kg (body weight) in saline) for hM4D(G_i_)-mediated inhibition and hM3D(G_q_)-mediated excitation experiments. Behavioral tests were performed after 1 h of administration of clozapine. For combined excitation experiments through hM3D(G_q_) together with peripheral sensory tests, the dosage of clozapine was measured by titration, and a final dose of 0.04 mg per kg (body weight) was applied. Combined behavioral tests were arranged after 2.5 h of administration of clozapine, where the effect of clozapine was considered subthreshold.

### Single-cell suspension preparation

Mice expressing a Cre-dependent tdTomato reporter (10–20 weeks old, both males and females) were killed by lethal overdose of isoflurane. The lumbar spinal cord was dissected and kept in freshly oxygenated artificial cerebrospinal fluid (ACSF) solution^22^ on ice. Spinal gray matter from the lumbar spinal dorsal horn was dissected and transferred to a plastic Petri dish with 2 ml of prewarmed digestion enzyme in ACSF solution (papain (25 U ml^–1^) and DNase I (55 U ml^–1^), Worthington Biochemical). For spinal dorsal horn neuronal atlas construction, each experiment included two to three mice (BAF53b-Cre*R26^Tom^). For labeled active ensembles or neurons from SNI mice, four mice were included for each suspension experiment. For suspension, spinal cord tissues were cut into pieces and triturated (ten times up and down) every 10 min (four times) using glass Pasteur pipettes (precoated with 0.5% bovine serum albumin). Cell suspensions were filtered through a 30-µm cell strainer (CellTrics, Sysmex) and washed with an additional 1.5 ml of ACSF and 0.5 ml of PBS. After spinning down (300*g* for 5 min at 4 °C) and resuspending with 2 ml of ACSF, gradient centrifugation with OptiPrep density gradient medium (5.4% iodixanol in ACSF; Sigma) was performed at 300*g* for 10 min at 4 °C. The cell pellet was resuspended with 4 ml of ACSF. SYTOX Blue (Invitrogen, Thermo Fisher Scientific) was added to stain dead cells. tdTomato^+^ and SYTOX Blue^–^ cells were sorted by fluorescence-activated cell sorting (FACS) on a BD FACSAria Fusion or BD FACSAria III at 4 °C. Cells were spun down and resuspended in a proper volume (~1,000 cells per µl) of ACSF solution.

### Nuclei isolation for snRNA-seq

Wild-type male mice (C57BL/6, 4 months old, *n* = 4) were allowed to survive for 30 min after heat stimulation. Wild-type control mice (3–4 months old, *n* = 4 males and 3 females) and mice 4 weeks after SNI (*n* = 4 males and 3 females) were killed by lethal overdose of isoflurane. Fresh lumbar spinal cord (L4–L6) was dissected, separated through ventral median fissure, snap-frozen on dry ice and kept at –80 °C until use. Spinal nuclei were isolated by Dounce homogenization in homogenization buffer (10 mM Tris (pH 8.0), 250 mM sucrose, 25 mM KCl, 5 mM MgCl_2_, 0.1 mM DTT, protease inhibitor cocktail, 0.5% RNase inhibitor, 0.1% Triton X-100 and 5 μg ml^–1^ transcriptional inhibitor actinomycin D) on ice, filtered with a 30-μm cell strainer twice and centrifuged (1,000*g* × 10 min, 4 °C). After removing debris by gradient centrifugation with 25–29% iodixanol (13,500*g* × 20 min, 4 °C), the pellet containing the nuclei was resuspended and incubated with NeuN-PE (1:250) on ice for 30 min. Nuclei were spun down (500*g* × 5 min, 4 °C), resuspended, stained with DAPI and sorted by FACS for DAPI^+^NeuN^+^ nuclei on a BD FACSAria Fusion sorter. Nuclei were concentrated to 500–1,000 nuclei per μl for snRNA-seq.

### Single-cell/single-nucleus gene expression 3′ sequencing

Sorted cells/nuclei were loaded onto a 10x Chromium Chip G to yield single-cell droplets with a v3 or v3.1 kit (10x Genomics). Reverse transcription, cDNA amplification and library construction were performed according to the user guide provided by the manufacturer. Pooled libraries were sequenced on an Illumina NovaSeq 6000 sequencing platform on an SP-100 flow cell with 91-bp sequencing into the 3′ end (5′ to 3′) of mRNAs at the National Genomics Infrastructure (SciLifeLab). Raw sequencing data were demultiplexed, converted into fastq format and aligned to the mouse reference mm10 using the STAR aligner (or cellranger count for SNI spinal nuclei samples) to generate the gene–cell matrices. Spinal nuclei samples from SNI experiments were sequenced on a 10B-300 flow cell, trimmed and aligned to the mouse reference with cellranger count from 10x Genomics.

### scRNA-seq/snRNA-seq data analysis

Gene expression matrices were imported into R (4.1.0–4.4.1) and analyzed with Seurat (4.0.6–5.1.0) with standard pipelines (Satijalab). To construct the neuronal atlas, cells were filtered out if they had fewer than 2,000 genes or a ratio of mitochondrial genes of more than 20%. To avoid possible overclustering, we chose an approach where the cells were clustered from the highest level of separation through the adjustment of dimensionality and resolution, followed by merging of transcriptionally highly similar clusters or separating functionally hybrid clusters. Non-neuronal cells were filtered out according to the expression of neuronal marker genes *Rbfox3* and *Snap25*. Finally, 27 clusters were produced for the spinal neuronal atlas with reduction = ‘pca’, dims = 1:25 and resolution = 0.5. Cluster-specific marker genes were identified with the FindAllMarkers() function. A Wilcoxon rank-sum test was selected to identify differentially expressed genes with at least an increased log_2_(fold change) of 0.25. Specific gene markers, including canonical and new markers, were selected from the list of differentially expressed genes for unbiased classified cell clusters.

For non-neuronal cells from SNI and control samples, individual cells were filtered out from the dataset (only protein-coding genes) if they had fewer than 1,000 genes, less than 4,000 unique molecular identifiers (UMIs) or a ratio of mitochondrial genes of more than 10%. The same pipeline described above was applied, where the dims (1:5) and resolution (0.4) were adjusted for oligodendrocytes and microglia.

For spinal nuclei from SNI and control samples, nuclei with more than 2,000 UMIs and a ratio of mitochondrial genes of less than 1% were kept for further analysis. For spinal nuclei from heat stimulation and control samples, nuclei with more than 4,000 UMIs and a ratio of mitochondrial genes of less than 2% were kept for further analysis, as described above.

### Cell identity classification/prediction

The classifier was built using scPred (v. 1.9.2), which was based on a low-dimensional representation of gene expression, with a mixture discriminant analysis as the underlying model. Both the reference and query data were normalized with the same normalization method. The query data were aligned to the reference data and classified using the pretrained models. All neurons with a maximum prediction score greater than or equal to 0.55 were assigned the label of the highest scoring cell type, and the unassigned cells were filtered out.

### Analysis with scCODA

The analysis of cell types between mechanical and heat ensembles was performed with scCODA, which is a Bayesian model for compositional single-cell data analysis^31^. The cutoff value for ‘false discovery rate’ was set as 0.2, and Ex15 was set as the reference cell type.

### Pseudobulk differential expression analysis

DESeq2 (ref. ^72^) was implemented here to perform pseudobulk differential expression analysis on a specific cell-type cluster (SNI versus control) with a standard workflow in R. The raw counts and metadata were extracted from the dataset containing SNI and control groups. Counts at the single-cell level were aggregated at the sample level for each cluster, and the corresponding metadata at the sample level were also generated. Quality control at the sample level was performed with principal component analysis and hierarchical clustering methods based on normalized and regularized log-transformed counts. DESeq2 differential expression analysis was performed, and dispersion estimates were plotted. The results for contrasted groups (SNI relative to control) were adjusted by shrinking the log_2_ (fold change) values using the apeglm method^73^. Results of differentially expressed genes (Wald test) were filtered with an adjusted *P* value of <0.01 and 50% increase or 50% decrease.

### SCENIC analysis

SCENIC^35^ was implemented here to explore the effect of SNI on cell status for each cell cluster in the RNA-seq data with standard pipelines (Aertslab). Coexpression networks were inferred by running GENIE3. GRNs (regulon) were built based on coexpression modules and transcription factor motif analysis (RcisTarget). Regulons were scored in cells with AUCell. Only nonextended regulons from the neuronal dataset were kept for regulon activity visualization. The Fos module score was produced with the list of genes in the Fos regulon through AddModuleScore() in R.

### Perturbation analysis

To identify cell types most affected by SNI, we performed a perturbation using the R package Augur^74^. Raw scRNA-seq data from SNI animals were processed, and cell labels were assigned from the reference data using scPred. The reference and SNI datasets were merged and filtered to include only protein-coding genes while also excluding any genes located on the Y chromosome. The data were then integrated with Seurat while removing batch effects. Augur was then run between control and SNI datasets for each cell type using default settings. As a negative control, we ran another round of Augur analysis on the same merged dataset with randomly shuffled treatment labels.

### RNA velocity

The M25 version (GRCm38.p6) from GENCODE was used as a reference for mouse sequencing data. According to the reference sequence, we generated the composite kallisto index of the separate fragments for spliced and unspliced transcripts. The spliced and unspliced counts of each cell were quantified following the kallisto bustools workflow for 10x scRNA-seq^75^. Cells were selected with the criteria of 2,000 expressed genes in each cell and at least 20 counts (both unspliced and spliced) for a gene. Overdispersed genes were normalized and log transformed. Thirty principal components were selected, and data were calculated via balanced k-nearest neighbor imputation at 30. Next, the dynamical model from scVelo^76^ was applied for estimating velocities. The embedding scatter plot was obtained from the UMAP generated by the Seurat analysis. For better visualization, the scaling factor in the Gaussian kernel around the grid point was set as 0.35, and the threshold for mass to be exhibited was set to above 2.

### Cross-species correlation analysis of GRNs

Cross-species correlation analysis of GRNs was conducted using the scCAMEL toolkit^59^, which was used for estimating cell-type similarity and integrating data across species. The SWAPLINE package within scCAMEL calculates probabilistic scores for cell types by training a neural network model on the most differentially expressed genes, excluding cell cycle-related genes. These genes are normalized and used for model training, with accuracy validated through *k*-fold cross-validation. For cross-species data integration, scCAMEL uses interpretable neural network learning. Each dataset is sequentially used as a reference for cell-type prediction in other datasets. All the training prediction results are merged for principal component analysis to identify key components, which are then used for illustrating cell-type similarities. Additionally, gene expression normalization aligns gene symbols across species, and transcription factor-related GRNs are identified by using GENIE3, facilitating further correlation analysis.

### Pain-like behavioral tests

Animals were habituated to the testing environment twice before behavioral experiments started. Stressed animals were not included during the testing day. The mechanical withdrawal threshold was tested in clear glass (8 cm in diameter) on a metal mesh floor and measured by logarithmically incremental stiffness of 0.04-, 0.07-, 0.16-, 0.40-, 0.60-, 1.0- and 2.0-g von Frey filaments (Stoelting) combined with an up–down method to assess tactile allodynia^71^. The cutoff of a 2.0-g hair was selected as the upper limit for testing. Heat sensitivity was assessed with a plantar test (Hargreaves method, IITC Life Science), and withdrawal latency was recorded^69^. A cold plantar test was performed with a dry ice pellet (9 mm, AGA AB) under the glass (the same settings of Hargreaves test), and withdrawal latency was recorded.

Paw withdrawal reflexes involve spinal cord local circuits, whereas nocifensive responses (affective-motivational responses), including paw shaking, lifting/guarding or licking (Supplementary Video 1), require forebrain regions (rostral to the mesencephalon) when the stimulation is removed^77,78^. To quantitatively scale pain responses, measures of nocifensive episodes or durations of nocifensive responses were applied. Nocifensive episode, which might contain different numbers or types of nocifensive responses, was used for quantifying responses to heat in the hot plate test where the stimulation was maintained for a fixed time. Mechanical-stimulated response was tested with a 2.0-g von Frey filament three times (successfully applied), and the average duration of nocifensive behaviors was calculated. Alternatively, mechanical-stimulated responses were tested with a 1.0-g von Frey filament in a combined excitation experiment, and the duration of nocifensive behaviors was recorded. Response to cooling stimulation was tested with a drop of acetone, and the duration of nocifensive behaviors was recorded^71^. Cold-stimulated response was tested with a drop of prechilled acetone on dry ice, and the duration of nocifensive behaviors was recorded^71^. Heat-stimulated response was tested by hot plate, and the duration of nocifensive behaviors was recorded for 3 min (46 °C), 90 s (48 °C) and 60 s (50 °C). The selection of temperatures for different experimental scenarios was based on the response severity (ranged from 46 to 50 °C). A hot plate test assessed with nocifensive response latency for pain sensitivity was also included. SNI-induced heat hypersensitivity was tested by a water drop (50 °C) applied to the lateral hind paw of animals on a mesh floor through a 20-ml syringe.

A pilot experiment was conducted on the captured mechanical ensemble, measuring behavior in response to a 2-g von Frey filament, hot plate and cold acetone in male and female mice. No differences were observed. Male mice were used for all remaining pain-like behavioral experiments. The experimental testers were blinded to animal groups during behavioral tests and quantification of videos.

### Tissue preparation and immunohistochemistry

Mice were deeply anesthetized with sodium pentobarbital (300 mg per kg (body weight) administered intraperitoneally; Apoteket) and perfused transcardially with freshly made 4% paraformaldehyde. The lumbar spinal cord was dissected, postfixed in fixative for 90 min at 4 °C and rinsed in 10% (wt/vol) sucrose containing 0.01% sodium azide (Merck) and 0.02% bacitracin (Sigma) for 2 day at 4 °C. All trimmed tissues were embedded with optimal cutting temperature cryomount (HistoLab), frozen with liquid carbon dioxide through sublimation, sectioned on a cryostat (NX70, Thermo Fisher Scientific) at a thickness of 20 μm for the spinal cord and stored at −20 °C until use.

Spinal cord sections were dried at room temperature for at least 30 min, pretreated with xylene twice for 10 min each to retain tissue morphology to the greatest extent and rehydrated with downgraded ethanol before antigen retrieval with Target Retrieval Solution (Agilent Dako). After blocking with 10% serum, primary rabbit anti-Fos (1:1,000; Santa Cruz) was added and incubated in a humid chamber at 4 °C for 2 days. Immunoreactivity was visualized using a TSAPlus Fluorescein kit (PerkinElmer)^79^. For double labeling, slides with c-Fos labeling or tdTomato reporter were rinsed in PBS for 20 min and incubated with primary antibody to PKCγ (1:200; Santa Cruz), TVA (1:200; from A. Leavitt, University of California, San Francisco) or SOX10 (1:400; Santa Cruz) over 48 h at 4 °C. After washing with PBS, samples were incubated with donkey anti-rabbit IgG (H + L) highly cross-adsorbed secondary antibody conjugated to Alexa Fluor 488, 555 or 647 (1:800; Invitrogen, Thermo Fisher Scientific) at room temperature for 2 h, rinsed with PBS for 30 min and counterstained with DAPI (Thermo Fisher Scientific) or propidium iodide (Sigma). Antibody information is provided in Supplementary Table 6.

### Tissue preparation and in situ hybridization (RNAscope)

Mice were killed by lethal overdose of isoflurane. The lumbar spinal cord and/or DRGs was dissected and snap-frozen on dry ice. Frozen tissues were thawed on ice briefly, trimmed, embedded with optimal cutting temperature cryomount, frozen with liquid carbon dioxide and sectioned on a cryostat at a thickness of 20 μm for the spinal cord and 12 μm for DRGs and stored at −80 °C before use. For the CTb-488 lateral parabrachial nucleus tracing experiment, fresh spinal cord was subjected to overnight immersion fixation in 4% paraformaldehyde (Histofix, Carl Roth) at 4 °C, followed by immersion in 10% sucrose for 2 days at 4 °C. The samples were processed the same as fresh frozen tissue. Immunostaining for mCherry (anti-RFP, 1:500; Rockland) and CTb (anti-CTb, 1:500; List labs) on these sections with Alexa Fluor dyes was performed as described above.

The RNAscope assay was performed according to the protocol provided with the RNAscope Multiplex Fluorescent Detection kit v2 (323110, ACDBio) with minor modifications, where the hydrogen peroxide treatment was skipped and protease III was used instead of protease IV. Probes included in this study were designed and provided by ACDBio, as listed in Supplementary Table 6.

### Microscopy and image processing

Representative images were acquired from a 1-Airy unit pinhole on an LSM700/LSM800/LSM900-Airy (Zeiss) or Leica Stellaris 5 confocal laser-scanning microscope. For projection images, orthogonal *z* stacks were acquired with a depth interval of 1 μm. Images were processed using ZEN software (Zeiss) or Fiji (ImageJ, NIH). Multipanel figures were assembled using Adobe Photoshop and Adobe Illustrator (Adobe Systems).

### Statistics

Nonparametric data are presented as median with interquartile range and were assessed by two-tailed Mann–Whitney test or Kruskal–Wallis test followed by post hoc analysis. For parametric data, data distribution was assumed to be normal, but this was not formally tested. Data are expressed as mean ± s.d. and were assessed by unpaired *t-*test (two-tailed) or ordinary one-way ANOVA followed by post hoc analysis using Prism 10 software (GraphPad), as specified in the figure legends. The criterion for statistical significance was *P* < 0.05.

### Reporting summary

Further information on research design is available in the Nature Portfolio Reporting Summary linked to this article.

## Online content

Any methods, additional references, Nature Portfolio reporting summaries, source data, extended data, supplementary information, acknowledgements, peer review information; details of author contributions and competing interests; and statements of data and code availability are available at 10.1038/s41593-025-01921-6.

## Supplementary information

Supplementary Information Supplementary Figs. 1–5.

Reporting Summary

Supplementary Tables 1–6 Supplementary Tables 1–6.

Supplementary Video 1 Nocifensive responses: shaking, lifting and licking.

## Source data

Source Data Fig. 1 Statistical source data.

Source Data Fig. 2 Statistical source data.

Source Data Fig. 3 Statistical source data.

Source Data Fig. 4 Statistical source data.

Source Data Fig. 5 Statistical source data.

Source Data Fig. 6 Statistical source data.

Source Data Extended Data Fig. 1 Statistical source data.

Source Data Extended Data Fig. 2 Statistical source data.

Source Data Extended Data Fig. 4 Statistical source data.

Source Data Extended Data Fig. 6 Statistical source data.

Source Data Extended Data Fig. 10 Statistical source data.

## Data Availability

Sequencing data from this study have been deposited in the Gene Expression Omnibus under accession code GSE253533. Additional resources for browsing gene expression of the spinal dorsal horn neuronal atlas are available at https://ernforslab.shinyapps.io/MouseDorsalHorn/. The mouse reference mm10 (GENCODE vM23/Ensembl 98, version 2020-A) is available at https://www.10xgenomics.com/support/software/cell-ranger/downloads/cr-ref-build-steps. Mouse and human spinal nuclei RNA-seq data from Kathe et al.^29^ and Yadav et al.^48^ used for comparison were downloaded from Gene Expression Omnibus via accession codes GSE184370 and GSE190442. Source data are provided with this paper.

## Code availability

Previously published code and software used for this study are cited and described in the Methods.
