# Supplementary material for: Neural ensembles that encode nocifensive mechanical and heat pain in mouse spinal cord
Source: Nat Neurosci. 2025 Mar 24;28(5):1012–23. doi: 10.1038/s41593-025-01921-6 (PMC12081300; doi:10.1038/s41593-025-01921-6)
Supplement: Supplementary file 1 — Supplementary Figs. 1–5. [file 41593_2025_1921_MOESM1_ESM.pdf]

nature neuroscience

Supplementary information

https://doi.org/10.1038/s41593-025-01921-6

Neural ensembles that encode nocifensive mechanical and heat pain in mouse spinal cord

In the format provided by the authors and unedited

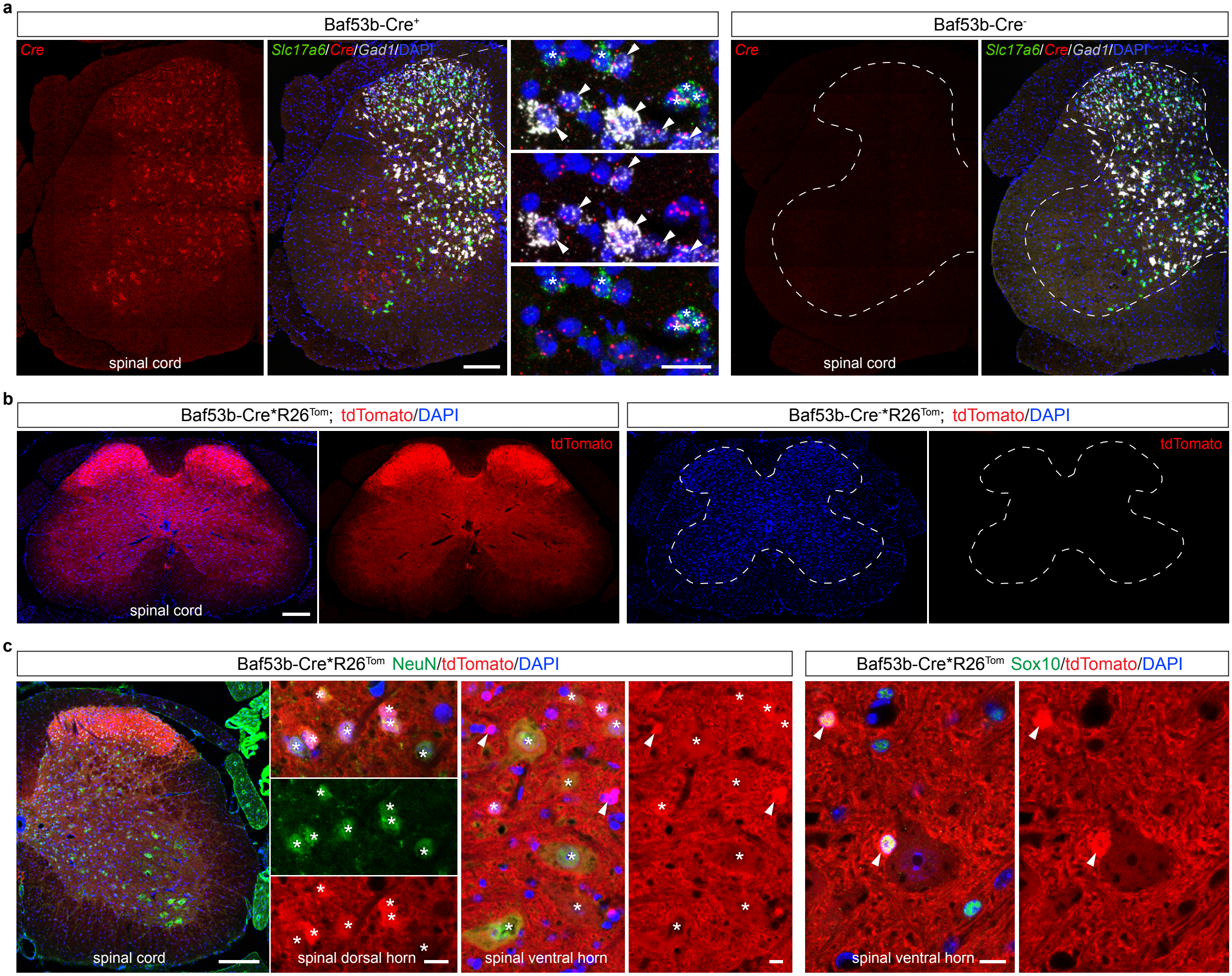

Supplementary Fig. 1 Validation of tdTomato expression in spinal cord from Baf53b-Cre*R26^{Tom} mice. a, Representative RNAscope images for triple labeling of *Cre* (BAF53), *Slc17a6* (excitatory) and *Gad1* (inhibitory) in spinal cord sections from Baf53b-Cre^{+} (n = 2) and Baf53b-Cre^{-} (n = 1) mice. *Cre* was detected in both excitatory and inhibitory neurons and most probably cholinergic neurons (negative for both *Slc17a6* and *Gad1*) from spinal ventral horn. No *Cre* signal was detected in spinal cord from Baf53b-Cre^{-} mice. Arrowheads indicate co-localization between *Cre* and *Gad1*. Asterisks indicate co-localization between *Cre* and *Slc17a6*. Dashed line shapes the spinal grey matter. **b,** Representative images of tdTomato expression in spinal cord from Baf53b-Cre*R26^{Tom} mice (n = 2). No tdTomato signal was observed in Baf53b-Cre negative mice (n = 2). **c,** Representative images of co-localization between tdTomato and NeuN (left panel, 4 µm projection image for high magnification) or Sox10 (right panel). Asterisks indicate co-localization between tdTomato and NeuN, whereas arrowheads indicate tdTomato^{+} cells negative for NeuN but positive for Sox10 from spinal ventral horn (n = 2 mice). DAPI was used for the nucleus counter staining. Scale bars: 200 µm for spinal cord overview in (**a-c**), 20 µm for the high magnification from rectangle with dashed line in (**a**) and 10 µm for high magnification in (**c**).

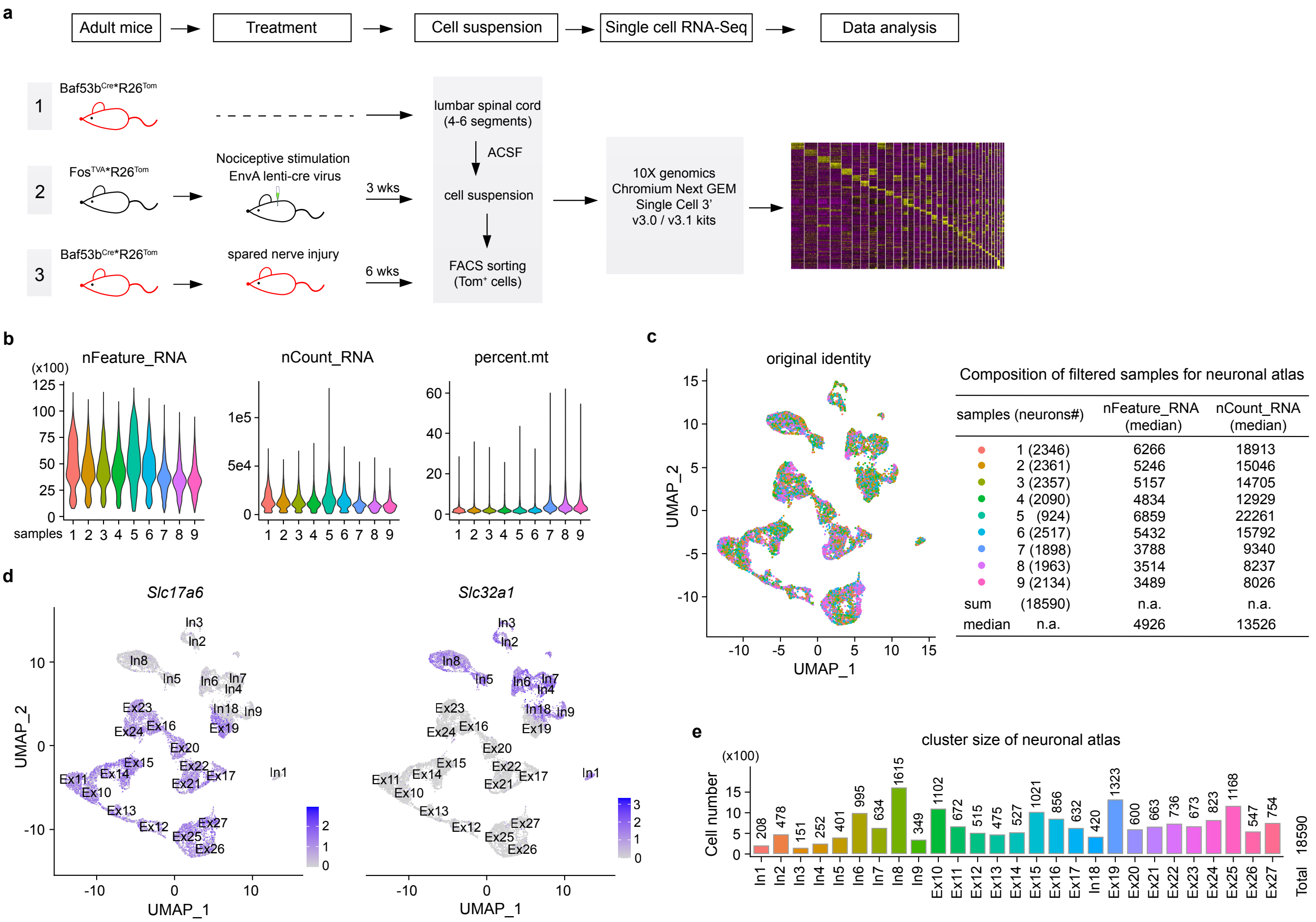

Supplementary Fig. 2 Workflow for single cell RNA-sequencing of neurons from adult mouse spinal cord and quality control of spinal neuronal atlas. a, Experimental workflow of single cell suspension preparations from different scenarios and RNA-Seq included in this study. **b**, Overview of sequencing quality including number of detected genes (nFeature_RNA), total reads of detected genes (nCount_RNA) and percentage of mitochondrial genes (percent.mt) for 9 samples (without filtering) from adult mice spinal cord. **c**, Left: UMAP of spinal neuronal atlas composed of cells from different samples. Right: table for numbers of neurons contributed from each sample to spinal dorsal horn neuronal atlas and their corresponding median for number of detected genes and total reads of detected genes. **d**, UMAP plots of excitatory *Slc17a6* (vGLUT2) and inhibitory *Slc32a1* (VGAT) marker gene expression. **e**, Size distribution (number of cells) of individual cluster across the atlas, which was composed of 18,590 neurons.

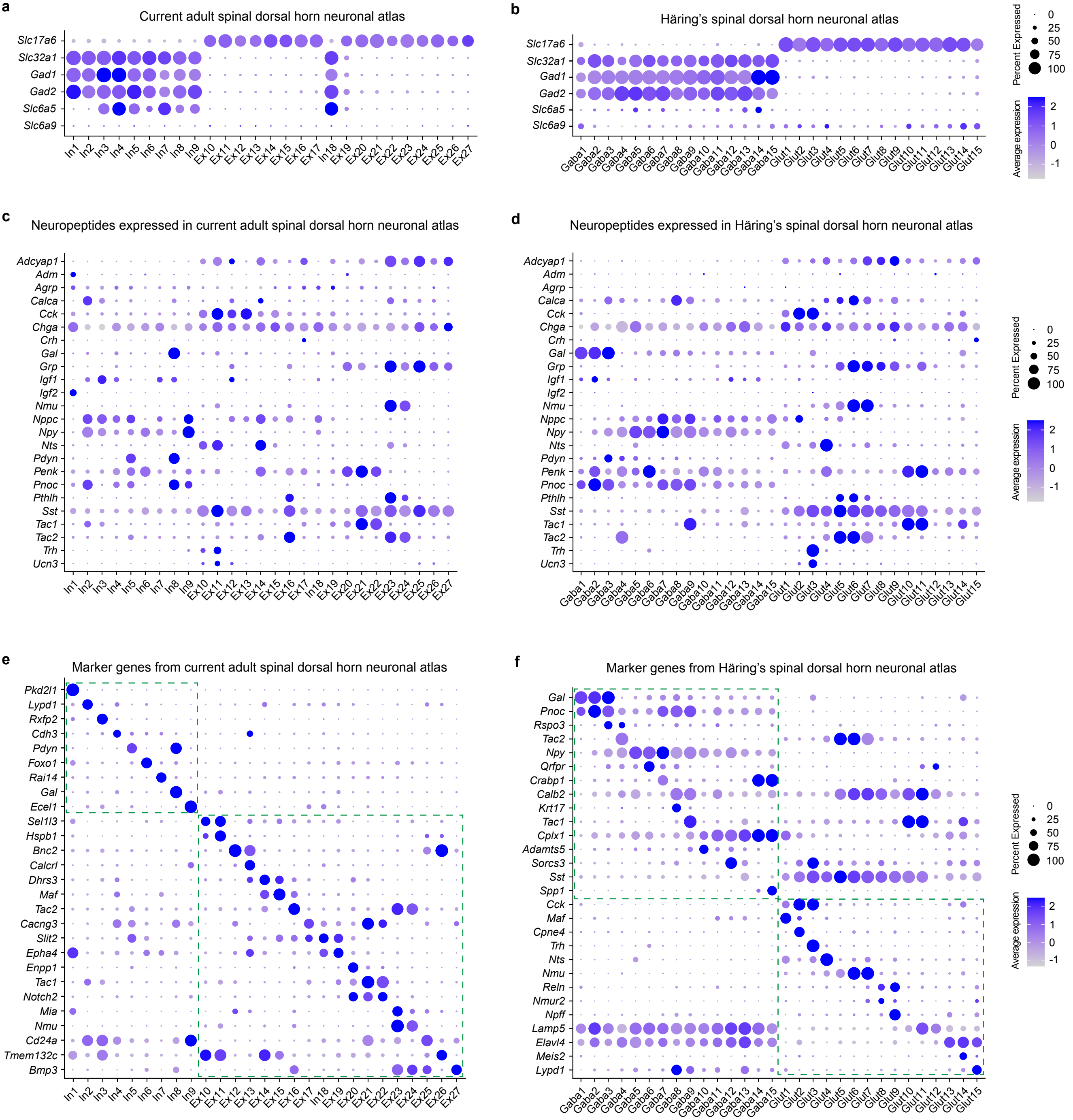

Supplementary Fig. 3 Comparison of expressions for excitatory/inhibitory genes, neuropeptides and lists of selected genes in current atlas and Häring's dataset. a and **b**, Dot plots show expression of *Slc17a6*, *Slc32a1*, *Gad1* (GAD67), *Gad2* (GAD65), *Slc6a5* (GlyT2, neurons) and *Slc6a9* (GlyT1, predominantly astrocytes). **c** and **d**, Dot plots show expression of a list of neuropeptides. **e**, Dot plot shows selected marker genes for neuronal clusters of current atlas. **f**, Dot plot shows published marker genes in Häring's dataset. Dashed line rectangles highlight the enriched gene expression for inhibitory and excitatory clusters in (**e** and **f**).

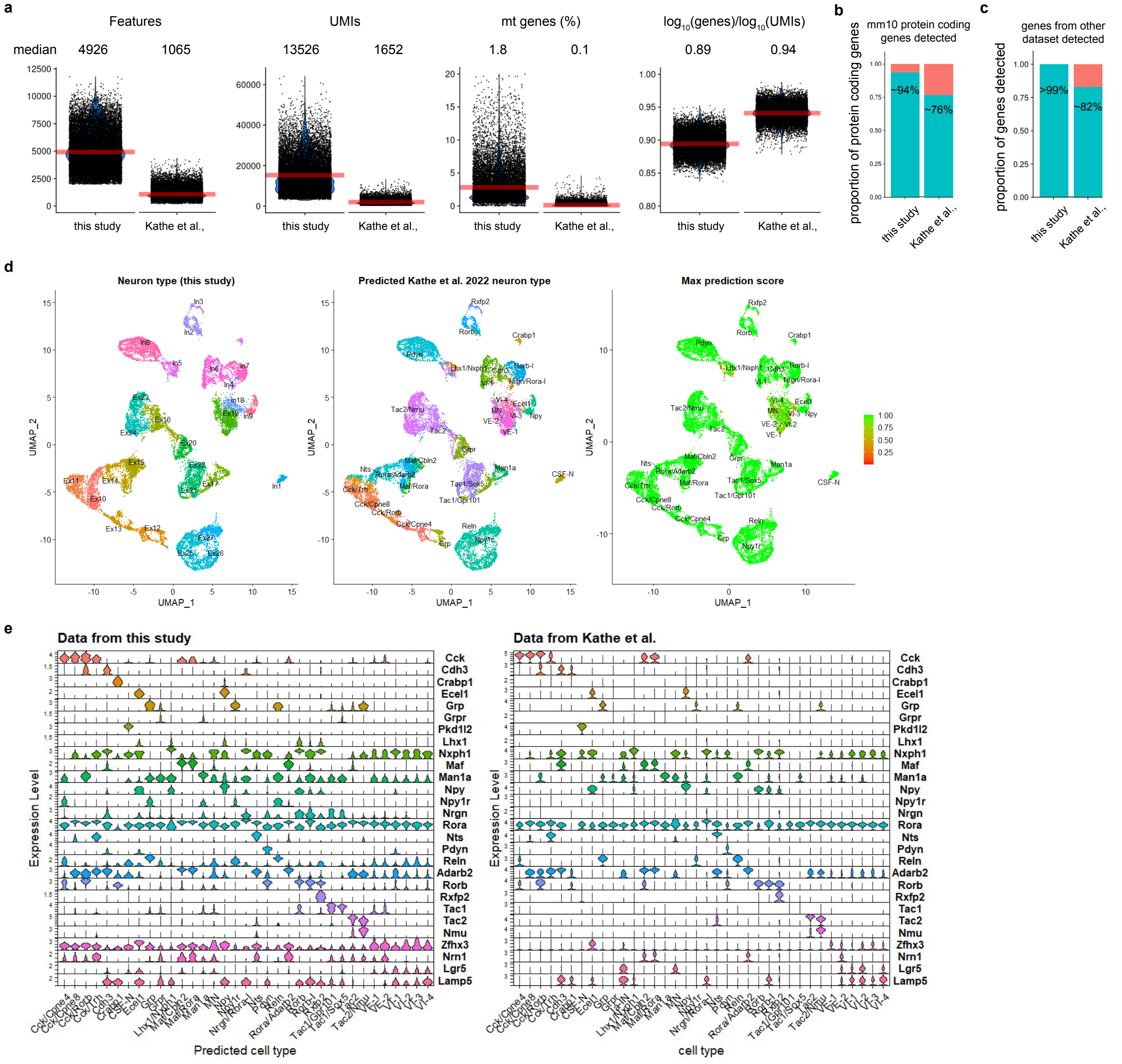

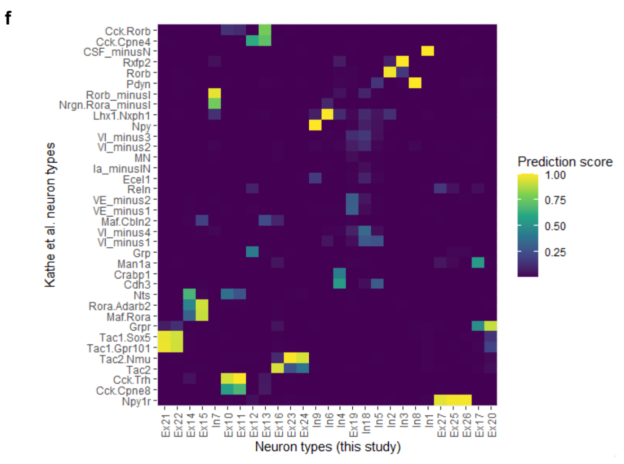

Supplementary Fig. 4 Comparison with Kathe et al. 2022 snRNA-seq dataset. a, Violin plots showing QC metrics for scRNA-seq data from current study and for snRNA-seq data from Kathe et al. study. Red horizontal lines correspond to median value (also indicated above each plot). **b**, Bar plots showing the proportion of protein-coding genes from the mouse genome (mm10) detected in the two datasets. **c**, Proportions of genes detected between the two datasets: (left) genes from current study in Kathe et al. and (right) vice versa. **d**, UMAPs for the current dataset showing: (left) cell types defined in the current study, (center) cell types predicted from Kathe et al. study and (right) maximum prediction score against each type for Kathe et al. **e**, Violin plots showing gene expression of main cell type markers from Kathe et al. in the current data (left) using predicted Kathe et al. labels and in the data from Kathe et al. (right) for comparison. **f**, Heatmap showing the mean prediction score for each cell type from current study against cell types from Kathe et al.

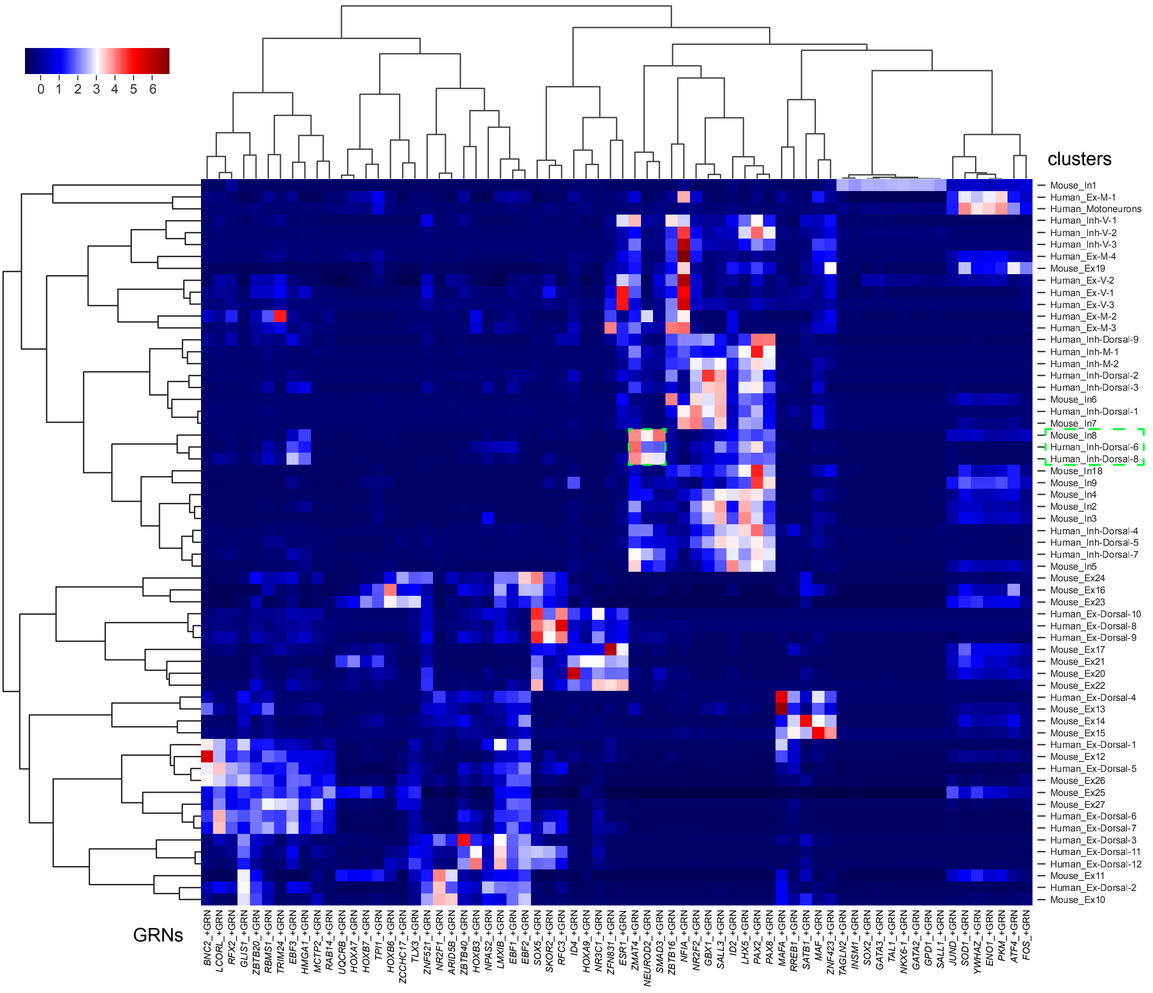

Supplementary Fig. 5 Conservation of In8 Gal⁺ population between mouse and human spinal cord. Heatmap with hierarchical clustering shows similar gene regulatory networks (GRNs) between mouse In8 and human Inh-Dorsal-6/8 (indicated by the green rectangle) according to Yadav's 2023 annotation.
