# Supplementary material for: Neural ensembles that encode nocifensive mechanical and heat pain in mouse spinal cord
Source: Nat Neurosci. 2025 Mar 24;28(5):1012–23. doi: 10.1038/s41593-025-01921-6 (PMC12081300; doi:10.1038/s41593-025-01921-6)
Supplement: Supplementary file 2 — Reporting Summary [file 41593_2025_1921_MOESM2_ESM.pdf]

nature portfolio

Corresponding author(s): Patrik Ernfors

Last updated by author(s): Jan 29, 2025

Reporting Summary

Nature Portfolio wishes to improve the reproducibility of the work that we publish. This form provides structure for consistency and transparency in reporting. For further information on Nature Portfolio policies, see our Editorial Policies and the Editorial Policy Checklist.

Statistics

For all statistical analyses, confirm that the following items are present in the figure legend, table legend, main text, or Methods section.

n/a	Confirmed	
☐	☒	The exact sample size (*n*) for each experimental group/condition, given as a discrete number and unit of measurement
☐	☒	A statement on whether measurements were taken from distinct samples or whether the same sample was measured repeatedly
☐	☒	The statistical test(s) used AND whether they are one- or two-sided<br>*Only common tests should be described solely by name; describe more complex techniques in the Methods section.*
☐	☒	A description of all covariates tested
☐	☒	A description of any assumptions or corrections, such as tests of normality and adjustment for multiple comparisons
☐	☒	A full description of the statistical parameters including central tendency (e.g. means) or other basic estimates (e.g. regression coefficient) AND variation (e.g. standard deviation) or associated estimates of uncertainty (e.g. confidence intervals)
☐	☒	For null hypothesis testing, the test statistic (e.g. *F*, *t*, *r*) with confidence intervals, effect sizes, degrees of freedom and *P* value noted<br>*Give P values as exact values whenever suitable.*
☒	☐	For Bayesian analysis, information on the choice of priors and Markov chain Monte Carlo settings
☒	☐	For hierarchical and complex designs, identification of the appropriate level for tests and full reporting of outcomes
☒	☐	Estimates of effect sizes (e.g. Cohen's *d*, Pearson's *r*), indicating how they were calculated

Our web collection on statistics for biologists contains articles on many of the points above.

Software and code

Policy information about availability of computer code

Data collection	Zen Black 2010/ Zen Blue v2.3 and v3.1 (Zeiss) and LAS X (Leica) for confocal images
Data analysis	R (v. 4.1.0/4.4.1), ImageJ (v. 2.0.0), Seurat (v. 4.0.6./5.1.0), SCENIC (v. 1.3.1), scPred (v. 1.9.2), scCODA (v. 0.1.8), DESeq2 (v. 1.42.1), Augur (v. 1.0.3), scCAMEL (v. 0.31b0), scVelo (v. 0.2.5), Prism 10<br>All data analysis were based on open source code and previous publications (see details in the methods part of this study).

For manuscripts utilizing custom algorithms or software that are central to the research but not yet described in published literature, software must be made available to editors and reviewers. We strongly encourage code deposition in a community repository (e.g. GitHub). See the Nature Portfolio guidelines for submitting code & software for further information.

Data

Policy information about availability of data

All manuscripts must include a data availability statement. This statement should provide the following information, where applicable:

- Accession codes, unique identifiers, or web links for publicly available datasets
- A description of any restrictions on data availability
- For clinical datasets or third party data, please ensure that the statement adheres to our policy

Accession code GSE253533 for sequence data and metadata (Data availability of the manuscript).
Interactive web-application for the mouse spinal dorsal horn neuronal atlas data (https://ernforslab.shinyapps.io/MouseDorsalHorn/).

Mouse reference, mm10 (GENCODE vM23/Ensembl 98, version 2020-A) is available through the link: https://www.10xgenomics.com/support/software/cell-ranger/downloads/cr-ref-build-steps.
Mouse and human spinal nuclei RNA sequencing data from references used for comparison were downloaded from Gene Expression Omnibus website with accession codes GSE184370 and GSE190442.

Research involving human participants, their data, or biological material

Policy information about studies with human participants or human data. See also policy information about sex, gender (identity/presentation), and sexual orientation and race, ethnicity and racism.

Reporting on sex and gender	n/a
Reporting on race, ethnicity, or other socially relevant groupings	n/a
Population characteristics	n/a
Recruitment	n/a
Ethics oversight	n/a

Note that full information on the approval of the study protocol must also be provided in the manuscript.

Field-specific reporting

Please select the one below that is the best fit for your research. If you are not sure, read the appropriate sections before making your selection.

☒ Life sciences ☐ Behavioural & social sciences ☐ Ecological, evolutionary & environmental sciences

For a reference copy of the document with all sections, see nature.com/documents/nr-reporting-summary-flat.pdf

Life sciences study design

All studies must disclose on these points even when the disclosure is negative.

Sample size	The sample size was determined according to our previous experience and publications (PMID: 28572455, PMID: 31416963, PMID: 29893745, PMID: 29686262). No statistical method was applied to predetermine sample size.
Data exclusions	For scRNA-Seq and snRNA-Seq data, cells with low quality were excluded according to the criteria of the quality control as described in the methods part of the manuscript.<br>For behavioral data, mice with signs of any unhealthy conditions or super active (stressed) during the testing day were excluded according to the predefined criteria.
Replication	The silencing experiments of ensembles for mechanical and heat in spinal cord in Figure 2 and the sequencing data for Figure 3 were repeated independently. Biological replicates (n) were indicated in figure legends.
Randomization	The animals for the experiments were randomized assigned.
Blinding	Single cell data collection and analysis were not blinded. Sample preparation, data quality control and analysis were not affected by blinding. Image collection for quantification and comparative analysis were performed blindly. Characterization of monosynaptic rabies virus traced neurons was not blinded for specific Cre mouse strains, but performed by different labs. Animal behavioral testers were blinding during the conduct of the test and quantification of videos.

Reporting for specific materials, systems and methods

We require information from authors about some types of materials, experimental systems and methods used in many studies. Here, indicate whether each material, system or method listed is relevant to your study. If you are not sure if a list item applies to your research, read the appropriate section before selecting a response.

Materials & experimental systems

n/a	Involved in the study
☐	☒ Antibodies
☒	☐ Eukaryotic cell lines
☒	☐ Palaeontology and archaeology
☐	☒ Animals and other organisms
☒	☐ Clinical data
☒	☐ Dual use research of concern
☒	☐ Plants

Methods

n/a	Involved in the study
☒	☐ ChIP-seq
☒	☐ Flow cytometry
☒	☐ MRI-based neuroimaging

Antibodies

Antibodies used

1. rabbit anti-c-Fos primary antibody (Santa Cruz Biotechnology, sc-52, Lot# D1411)
2. rabbit anti-PKCg primary antibody (Santa Cruz Biotechnology, sc-211, Lot# D0414)
3. rabbit anti-TVA primary antibody (home-made)
4. mouse anti-NeuN primary antibody (Millipore, clone A60, MAB377, Lot# 3713321)
5. goat anti-Sox10 primary antibody (Santa Cruz, sc-17342, Lot# K1815)
6. goat anti-CTb primary antibody (List labs, #703, Lot# 7032A12)
7. rabbit anti-RFP primary antibody (Rockland, #600-401-379, Lot# 42872)
8. Alexa Fluor 488 donkey anti-goat IgG(H+L) (Life Technologies, REF A11055, Lot# 1369678)
9. Alexa Fluor 555 donkey anti-rabbit IgG(H+L) (Invitrogen, REF A31572, Lot# 2339822)
10. Alexa Fluor 647 donkey anti-goat IgG(H+L) (Life Technologies, REF A21447, Lot# 1739289)
11. Alexa Fluor 647 donkey anti-mouse IgG(H+L) (Invitrogen, REF A31571, Lot# 1984047)

Validation

1. Fos antibody (PMID: 28572455), 2. PKCg antibody (PMID: 24616509), 3. TVA antibody (PMID: 27974160), 4. NeuN antibody (PMID: 28860091), 5. Sox10 antibody (PMID: 31416963) 6. CTb antibody (PMID: 12576182) 7. RFP antibody (PMID: 22121118)

Animals and other research organisms

Policy information about studies involving animals; ARRIVE guidelines recommended for reporting animal research, and Sex and Gender in Research

Laboratory animals

Mice strains C57BL/6N, Fos-2A-dsTVA (027831), Rosa26-Tom (007909), Rosa26-RC::FPDi (029040), Rosa26-DTA (009669), Actinb-FLPe (003800), Actl6b-Cre (027826), Npy-Cre (027851) from JAX and Gal-Cre (KI87) from GENSAT were included in this study. Mice used for RNA sequencing, strain characterization and tracing experiments were 2-4 months old. Mice used for behavioral experiments experiments were 3-8 months old.

Wild animals

This study did not include wild animals.

Reporting on sex

Both males and females were included in the study. Behavioral data presented in the study were conducted using male mice.

Field-collected samples

No field collected samples were included in this study.

Ethics oversight

The animal experiments were approved by the Stockholm and Uppsala Ethical Committees, which were organized under the Swedish Board of Agriculture.

Note that full information on the approval of the study protocol must also be provided in the manuscript.

Plants

Seed stocks

Report on the source of all seed stocks or other plant material used. If applicable, state the seed stock centre and catalogue number. If plant specimens were collected from the field, describe the collection location, date and sampling procedures.

Novel plant genotypes

Describe the methods by which all novel plant genotypes were produced. This includes those generated by transgenic approaches, gene editing, chemical/radiation-based mutagenesis and hybridization. For transgenic lines, describe the transformation method, the number of independent lines analyzed and the generation upon which experiments were performed. For gene-edited lines, describe the editor used, the endogenous sequence targeted for editing, the targeting guide RNA sequence (if applicable) and how the editor was applied.

Authentication

Describe any authentication procedures for each seed stock used or novel genotype generated. Describe any experiments used to assess the effect of a mutation and, where applicable, how potential secondary effects (e.g. second site T-DNA insertions, mosiacism, off-target gene editing) were examined.
